# Mosaic and mixed HIV-1 glycoprotein nanoparticles elicit antibody responses to broadly neutralizing epitopes

**DOI:** 10.1371/journal.ppat.1012558

**Published:** 2024-10-03

**Authors:** Mitch Brinkkemper, Gius Kerster, Philip J. M. Brouwer, Andy S. Tran, Jonathan L. Torres, Roos A. Ettema, Haye Nijhuis, Joel D. Allen, Wenwen Zhu, Hongmei Gao, Wen-Hsin Lee, Tom P. L. Bijl, Jonne L. Snitselaar, Judith A. Burger, Ilja Bontjer, Wouter Olijhoek, Rashmi Ravichandran, Marielle J. van Breemen, Iván Del Moral-Sánchez, Ronald Derking, Kwinten Sliepen, Gabriel Ozorowski, Max Crispin, David C. Montefiori, Mathieu Claireaux, Andrew B. Ward, Marit J. van Gils, Neil P. King, Rogier W. Sanders

**Affiliations:** 1 Amsterdam UMC, location University of Amsterdam, Department of Medical Microbiology and Infection prevention, Amsterdam, The Netherlands; 2 Amsterdam institute for Infection and Immunity, Infectious diseases, Amsterdam, The Netherlands; 3 Department of Integrative Structural and Computational Biology, The Scripps Research Institute, La Jolla, California, United States of America; 4 School of Biological Sciences, University of Southampton, Southampton, United Kingdom; 5 Duke Human Vaccine Institute and Department of Surgery, Duke University Medical Center, Durham, North Carolina, United States of America; 6 Department of Biochemistry, University of Washington, Seattle, Washington, United States of America; 7 Institute for Protein Design, University of Washington, Seattle, Washington, United States of America; 8 Department of Microbiology and Immunology, Weill Medical College of Cornell University, New York, New York, United States of America; National Institute for Communicable Diseases, SOUTH AFRICA

## Abstract

An effective human immunodeficiency virus 1 (HIV-1) vaccine will most likely have to elicit broadly neutralizing antibodies (bNAbs) to overcome the sequence diversity of the envelope glycoprotein (Env). So far, stabilized versions of Env, such as SOSIP trimers, have been able to induce neutralizing antibody (NAb) responses, but those responses are mainly strain-specific. Here we attempted to broaden NAb responses by using a multivalent vaccine and applying a number of design improvements. First, we used highly stabilized SOSIP.v9 trimers. Second, we removed any holes in the glycan shields and optimized glycan occupancy to avoid strain-specific glycan hole responses. Third, we selected five sequences from the same clade (B), as we observed previously that combining Env trimers from clade A, B and C did not improve cross-reactive responses, as they might have been too diverse. Fourth, to improve antibody (Ab) responses, the Env trimers were displayed on two-component I53-50 nanoparticles (NPs). Fifth, to favor activation of cross-reactive B cells, the five Env trimers were co-displayed on mosaic NPs. Sixth, we immunized rabbits four times with long intervals between vaccinations. These efforts led to the induction of cross-reactive B cells and cross-reactive binding Ab responses, but we only sporadically detected cross-neutralizing responses. We conclude that stabilized HIV-1 Env trimers that are not modified specifically for priming naive B cells are unable to elicit strong bNAb responses, and infer that sequential immunization regimens, most likely starting with specific germline-targeting immunogens, will be necessary to overcome Env’s defenses against the induction of NAbs. The antigens described here could be excellent boosting immunogens in a sequential immunization regimen, as responses to bNAb epitopes were induced.

## Author summary

The overall consensus in the HIV-1 vaccine field is that an effective vaccine should be able to elicit broadly neutralizing responses. In this study we aimed at inducing a cross-neutralizing antibody response using stabilized HIV-1 envelope trimers. We applied a multitude of state-of-the-art antigen design approaches to achieve this goal. Despite these efforts, our immunogens did not induce cross-neutralizing responses efficiently. In-depth analysis of the antibody responses, and antigen-specific B cells elicited by the vaccinations revealed interesting details that can help guide future vaccine design. Presenting the mature envelope proteins on a mixture of monovalent nanoparticles, or co-displayed on a mosaic nanoparticle, induced different amounts of cross-reactive cells. Furthermore, we found that the immunogens used in this study were in some cases able to induce antibody responses to broadly neutralizing epitopes, indicating that these immunogens could be useful in alternative vaccination strategies.

## Introduction

NAbs against HIV-1 have a single target on the virion, Env [1]. Env interacts with the cluster of differentiation 4 (CD4) receptor, and the C-C Motif Chemokine Receptor 5 (CCR5) or C-X-C chemokine receptor type 4 (CXCR4) co-receptors on target cells to facilitate fusion of the viral and host cell membranes. Env is a trimer of heterodimers which consist of the transmembrane gp41 and the surface gp120 glycoproteins. Upon interaction with its receptors, Env switches from a pre- to postfusion conformation to bridge and fuse the viral and cell membranes [2,3]. Most of the epitopes targeted by NAbs are present on prefusion Env, but HIV-1 has evolved multiple strategies to protect Env from NAb recognition. First, enormous sequence diversity, caused by a rapid mutation rate, prevents NAbs from recognizing more than only one or a few strains. The Env amino acid sequence can differ up to 35% between circulating strains [4]. Second, Env is surrounded by a dense glycan shield that acts as a barrier. This barrier protects the protein surface from immune recognition. Breaches in the glycan shield can expose neutralizing epitopes, but NAbs targeting these epitopes are usually strain-specific [5,6]. Third, Env is conformationally dynamic and unstable. Instability can cause shedding of the gp120 subunit, which creates neo-epitopes that are not targeted by NAbs. Also, Env can assume multiple conformations that expose non-neutralizing epitopes [7,8]. In this study we describe an HIV-1 subunit vaccine designed to overcome many of these hurdles.

During infection, HIV-1 continuously escapes from NAb recognition by mutating its Env, while mutated Env triggers new rounds of affinity maturation in B cells. In a small subset of individuals, the coevolution of viral Env and the immune system eventually results in the elicitation of broadly neutralizing antibodies (bNAbs) [9–12]. These bNAbs can neutralize most of the circulating strains. An effective HIV-1 vaccine will likely have to induce bNAbs to overcome Env’s sequence diversity. The first hurdle to overcome when designing a subunit HIV-1 Env vaccine is Env’s instability. Multiple Env designs have solved this problem by introducing stabilizing mutations. The SOSIP.664 design employs an optimized cleavage site between gp41 and gp120, an intermolecular disulfide bond to prevent gp120 dissociation, a proline substitution to maintain gp41 in the pre-fusion confirmation, and a truncation at position 664 to solubilize the protein [13,14]. Starting with the native-like BG505 SOSIP.664 trimer, this design has been applied to other genotypes and mutations were added to further increase stability, antigenicity, and improve production. More stable versions of the SOSIP design have been shown to induce improved NAb titers and increase heterologous Ab responses in animal models [15–17]. Here, we used the SOSIP.v9 design, the latest and most stable design developed in our group [17]. In addition to the modifications listed for the SOSIP.664 design, SOSIP.v9 contains, among other modifications, two additional gp120-gp41 disulfide bonds, one of which links the protomers together.

Previous studies have demonstrated that highly stable SOSIP Env trimers can elicit NAb responses, but these are mainly strain-specific, with limited neutralization breadth [18,19]. Multivalent vaccine formulation might lead to broadening of these immune responses. Multivalent vaccines might induce cross-reactive Ab responses and/or multiple independent strain-specific responses, with the degree of antigenic diversity probably being an important contributing factor in this process. We previously observed that combining Env trimers based on sequences from HIV-1 clade A, B and C viruses predominantly induced independent NAb responses, not cross-reactive responses [20]. We hypothesized then that more closely related immunogens, such as those derived from the same clade, might be better suited for the induction of cross-reactive responses. Therefore, in this study, we focused on immunogens derived from HIV-1 clade B.

The immunogenicity of Env trimers is highly dependent on their glycan shields, which cover large parts of the protein surface [21]. Indeed, SOSIP trimer vaccination studies have shown that immune responses are usually directed toward holes in the glycan shield [6]. These epitopes are often immunodominant and can direct the immune response away from more desired epitopes. It has been postulated that Envs with a dense glycan shield might drive the NAb response toward less immunogenic but more conserved epitopes [22,23]. To avoid strain-specific glycan hole responses, we selected Env trimers with dense glycan shields [24,25], and, where necessary we optimized them by reintroducing any missing conserved NxS potential N-linked glycosylation site (PNGS) sequences. Furthermore, PNGS were replaced by NxT motifs where appropriate, to maximize PNGS occupancy [26].

The Ab responses induced by HIV-1 Env immunogens are notoriously weak compared to those induced by (glyco)proteins from other viruses [27,28]. Multimeric antigen presentation is a well-established strategy for enhancing humoral immune responses. NP antigen display can aid multiple immune processes, including lymph node trafficking, antigen retention on follicular dendritic cells, and B cell activation through B cell receptor cross-linking [29–31]. Indeed, displaying SOSIP Env trimers on NPs improved NAb responses against the respective HIV-1 strains [32,33]. Here, we utilized the I53-50 two-component NP platform, which allows for quality-controlled production of SOSIP Env trimers before NP assembly [34]. Two-component NPs also facilitate co-display of diverse antigens to promote interaction with cross-reactive B cells, resulting in improved protective and/or heterologous NAb responses against influenza and hepatitis C virus in animal models [35–37]. Accordingly, the five SOSIP Env trimers we selected were co-displayed on I53-50 NPs to maximize the chances to induce cross-reactive immune responses.

Considering the above arguments, in this study, we immunized rabbits with a mosaic mixture of five, highly stabilized, highly glycosylated Env trimers based on HIV-1 clade B viruses presented on I53-50 NPs. We observed Ab responses to bNAb epitopes, indicating that the antigens used here are capable of eliciting Ab responses to desired targets. However, this did not lead to cross-neutralizing responses and even the autologous NAb responses were not strong.

## Results

### SOSIP.v9 constructs based on five clade B sequences yielded highly stable trimers

**Fig 1:**
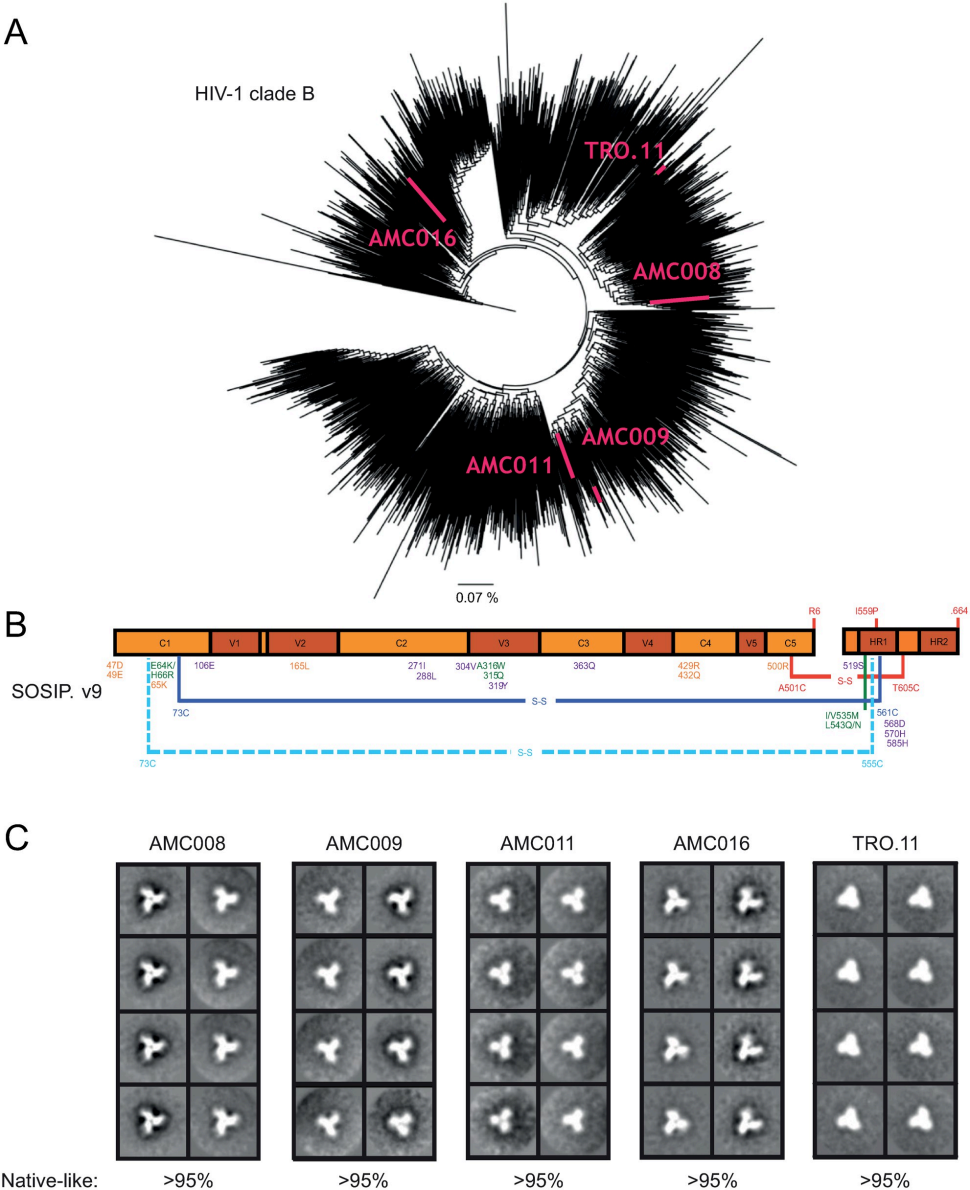
Clade B SOSIP.v9 proteins. (A) Phylogenetic tree of HIV-1 clade B Env sequences. To make the tree, 2355 sequences were collected from the Los Alamos database. The sequences used in the study are indicated in pink. The line presents 0.07% difference. (B) Linear schematic of the SOSIP.v9 construct. Amino acid substitutions are indicated in different colors based on where they were first described. SOSIP.664 mutations are shown in red [40]. SOSIP.v4 mutations are indicated in green [15]. SOSIP.v5 mutations are indicated in dark blue [16]. SOSIP.v6 mutations are shown in light blue [16]. TD8 mutations are shown in yellow [38]. MD39 mutations are shown in purple [39]. The lines indicate intra-protomer disulfide bonds and the dashed lines indicate inter-protomer disulfide bonds. (C) 2D class-average negative-stain EM images of the five clade B SOSIP proteins. The percentage of native-like trimers found in each sample is indicated.

We selected five HIV-1 clade B sequences based on their phylogenetic coverage across clade B, including AMC008, AMC009, AMC011 and AMC016 derived from the Amsterdam Cohort Studies and TRO.11 retrieved from an infected individual in Italy (Fig 1A). The similarities between the selected Env sequences, and BG505 Env (clade A) and ZM197M Env (clade C), at amino acid level are shown in S1 Table. The similarity between the clade B sequences was ~85%, while the similarity between de clade B sequences and BG505 and ZM197M were ~73%. To ensure maximal stabilization of the Env trimers we used the SOSIP.v9 design (Fig 1B) [17]. The modifications in this design include the previously described SOSIP.v6 mutations [16], which includes an inter-protomer disulfide bond, seven of the previously described ‘TD8’ mutations [38], and nine of the substitutions in the ‘MD39’ SOSIP design [39]. These modifications have been shown to improve yields, reduce aggregation and dissociation to dimers and monomers, increase stability, and improve the antigenic profile of BG505 SOSIP trimers [17].

Furthermore, to avoid immunodominant strain-specific Ab responses to glycan holes [6,41], we attempted to fill in any missing glycans in the glycan shields of these Env proteins. The guiding hypothesis was that by minimizing the induction of potentially distractive strain-specific responses, the induction of cross-reactive responses might be improved. Glycan holes were identified using the Los Alamos glycan shield mapping tool (S1 Fig) [23]. These analyses showed that AMC009, AMC011, AMC016 and TRO.11 Envs inherently have dense glycan shields with no apparent holes (no glycan holes >300 Å^2^) [41]. We identified a hole of >1,000 Å^2^ in the glycan shield of AMC008 around position N234, where the AMC008 sequence lacks a PNGS. Accordingly, a PNGS motif (NGT) was introduced at this position. Moreover, NxS motifs were replaced with NxT motifs where appropriate to improve occupancy and thus remove glycan holes stemming from NxS underoccupancy [26]. Our strategy did not address the large glycan free area at the bottom of soluble Env trimers [42], but we expected this to be of lesser concern when displaying the trimers on NPs.

The SOSIP genes were cloned into an expression vector and the constructs were expressed in HEK293F cells and purified using PGT145 or PGT151 affinity chromatography, followed by size exclusion chromatography (SEC) (S2A Fig). The detection of a gp120 band in reducing SDS-PAGE analysis showed that the SOSIP proteins were fully cleaved between the gp41 and gp120 subunits (S2B Fig), and non-reducing SDS-PAGE showed a single slow-migrating species consistent with three gp120/gp41 protomers held by inter-protomer disulfide bonds, presumably between 49C and 555C (S2C Fig) [16]. Blue Native-PAGE analysis revealed the formation of trimeric species (S2D Fig). Dynamic light scattering (DLS) experiments showed that, similar to other SOSIP trimers [15,32,33], the five SOSIP.v9 proteins were monodisperse and had hydrodynamic radii (*R*_h_) of 72–74 Å (Table 1), while negative-stain electron microscopy (nsEM) confirmed that the preparations consisted for >95% of native-like trimers with the typical propeller shape (Fig 1C).

**Table 1:** DSF and DLS of SOSIP.v9 proteins and SOSIP-I53-50 NPs.

	Thermostability (DSF)	Morphology (DLS)		
	*T*_m_ (°C)	*R*_h_ (Å)	*P*_d_ (%)	Mass (%)
AMC009 SOSIP.v5.2	70.0	n.d.	n.d.	n.d.
AMC011 SOSIP.v5.2	68.5	n.d.	n.d.	n.d.
AMC008 SOSIP.v9	76.6	72	10.5	100
AMC009 SOSIP.v9	79.1	73	12.4	100
AMC011 SOSIP.v9	78.9	73	22.9	100
AMC016 SOSIP.v9	81.5	73	16.6	98.3
TRO.11 SOSIP.v9	76.5	74	17.4	100
AMC008 SOSIP-I53-50 NP	n.d.	264	17.8	100
AMC009 SOSIP-I53-50 NP	n.d.	261	15.6	100
AMC011 SOSIP-I53-50 NP	n.d.	253	14.7	99.3
AMC016 SOSIP-I53-50 NP	n.d.	246	16	93.4
TRO.11 SOSIP-I53-50 NP	n.d.	252	15	100
Mosaic SOSIP-I53-50 NP	n.d.	292	27.2	99.0

Differential scanning fluorimetry (DSF) was used to determine the thermostability of the SOSIP.v9 proteins. The five SOSIP.v9 proteins were highly thermostable with midpoints of thermal denaturation or melting temperatures (*T*_m_) between 76.6°C and 81.5°C. For comparison, *T*_m_ values of the AMC009 and AMC011 SOSIP.v5.2 proteins were 70.0°C and 68.5°C, respectively, very similar to previous results obtained using differential scanning calorimetry (DSC) [24], but ~10°C lower than the corresponding SOSIP.v9 proteins (Table 1 and S3 Fig).

A panel of HIV-1 bNAbs and non-NAbs was used to assess antigenicity of the SOSIP.v9 constructs using an enzyme-linked immunosorbent assay (ELISA) (S4 Fig). Quaternary structure-specific bNAbs PGT145 and PGT151 interacted strongly with all five proteins, but quaternary-specific bNAb PG9 showed weak binding. The 2G12, PG16, b12, VRC01 and 35022 bNAbs showed moderate to strong binding to all constructs with the exception of b12, which did not bind to TRO.11. CD4 binding-site non-NAb F105 interacted very weakly or not at all with these SOSIP proteins. Overall, we conclude that the five clade B SOSIP.v9 trimers have appropriate antigenic structures, presenting multiple bNAb epitopes. Next, we tested a panel of bNAb precursors to assess whether these could bind the clade B SOSIP.v9 proteins (S5 Fig). For comparison we also included BG505 SOSIP.v8.1 and BG505 SOSIP.v8.1 GT1.1. BG505 SOSIP.v8.1 GT1.1 was specifically designed to bind bNAb precursors [43,44]. The bNAb precursors we tested, germline (gl)-VRC01, gl-12A12, gl-3BNC60, and gl-PG9, bound the SOSIP.v9 proteins very minimally or not at all. As expected, gl-VRC01, gl-12A12, and gl-PG9, did bind BG505 SOSIP.v8.1 GT1.1, while gl-3BNC60 did not. BG505 SOSIP.v8.1 and BG505 SOSIP.v8.1 GT1.1 both bound gl-PG9 equally, consistent with previous studies [43].

We next assessed the density and composition of the glycan shields. The SOSIP.v9 proteins were digested with trypsin, chymotrypsin, and alpha lytic protease and the peptide and glycopeptide pools were analyzed by liquid chromatography-mass spectrometry (LC-MS), which enabled the determination of the site-specific occupancy of PNGS and the composition of the attached N-linked glycans (S6 Fig). For some sites, glycopeptides of sufficient quality were not obtained and the occupancy of these sites could not be determined. Glycan occupancy of PNGS on gp120 was high, with only a few sites being substantially under occupied (<75% occupancy). These included N141 (on the AMC008 trimer), N150b (TRO.11), N156 (AMC011), N339 (AMC008, AMC009, AMC016), and N362 (AMC011, TRO.11). The occupancy on gp41 was somewhat lower, but higher than on previously studied SOSIP trimers, including earlier versions of AMC009, AMC011 and AMC016 trimers, that did not have NxS to NxT changes to enhance occupancy [24,26,41]. The predicted glycan hole on AMC008 SOSIP at position N234 was effectively filled by inserting the N234 PNGS, as it was fully occupied.

Glycan composition was, overall, similar across all clade B SOSIP proteins and consistent with the composition on other SOSIP trimers [24,26,41], with an abundance of high-mannose glycans on both gp120 and gp41. This includes the key glycan sites located in the intrinsic mannose patch that form the epitopes for V3-N332 bNAbs, and include the glycan at N332. Furthermore, the trimer-associated mannose patch was conserved at the apex of the trimer, specifically around N156 and N160. The glycans at N88 and N355/N356, as well as those located in the V1, V2, V5 and gp41 domains were predominantly occupied by complex glycans, and this observation is again consistent with previous observations for SOSIP trimers. Interestingly, the glycan at N276 was oligomannose on most trimers [24,26,41], but predominantly complex on AMC008 trimers. We conclude that the five SOSIP.v9 trimers have dense glycan shields in comparison with prototype SOSIP trimers such as the BG505 SOSIP.664 trimer [26,41]. From here on we refer to these SOSIP.v9 trimers as SOSIP trimers.

### Five SOSIP.v9 trimers can be displayed and co-displayed on I53-50 nanoparticles

**Fig 2:**
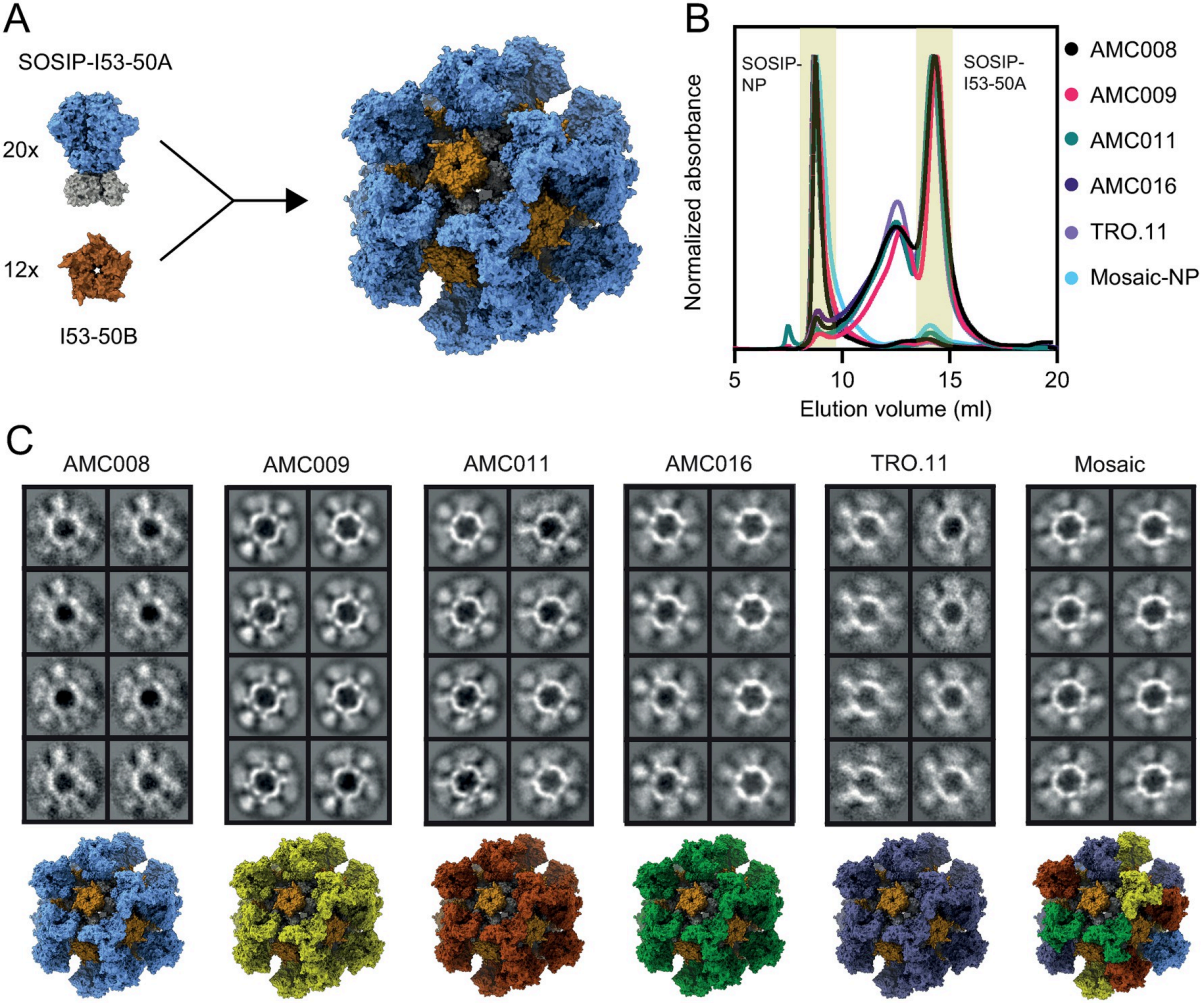
I53-50 NPs displaying SOSIP.v9 proteins. (A) Schematic of SOSIP-I53-50 NP assembly. The SOSIP is indicated in blue (PDB: 6X9V), I53-50A in gray (PDB: 6P6F), and I53-50B in brown (PDB: 6P6F). (B) Size exclusion chromatogram of the five SOSIP-I53-50A proteins, and the assembled monovalent and mosaic NPs. The collected fractions are indicated in yellow. The graph is an overlay of multiple runs. (C) 2D class-average negative-stain EM images of the assembled SOSIP-I53-50 NPs. Depictions of the different particles, including mosaic, are shown below. SOSIPs based on different strains are shown in different colors (PDBs: 6X9V and 6P6F).

I53-50 NPs consist of 20 trimeric components (I53-50A or variants thereof) and 12 pentameric components (I53-50B.4PT1) (Fig 2A). The SOSIPs were genetically fused to I53-50A. SOSIP-I53-50A proteins were expressed in HEK293F cells and purified using affinity chromatography and SEC as described above (Fig 2B). Reducing SDS-PAGE analysis showed that the SOSIP-I53-50A proteins were efficiently cleaved between the gp41 and gp120 subunits (S7A Fig), and non-reducing conditions showed full formation of inter-protomer disulfide bonds (S7B Fig). We compared the glycosylation of the SOSIP-I53-50A trimer with that of the parental SOSIP trimers described above (S8 Fig). The coverage of glycopeptides of the SOSIP-I53-50A proteins was higher than for the SOSIP proteins, resulting in data on additional glycans. For example, while N241 was only observed for AMC016 SOSIP, it was observed for all five SOSIP-I53-50A proteins, revealing that it is often inefficiently occupied, possibly because of its close proximity to the fully occupied N234 site. When comparing the glycosylation of the SOSIP and SOSIP-I53-50A constructs, the patterns were overall very similar, but two substantial differences were observed (S8 Fig). First, the extension of the C terminus through addition of the I53-50A component caused an increase of PNGS occupancy in gp41. For example, N611 occupancy was increased on AMC008 and AMC009 SOSIP-I53-50A compared to SOSIP, while N637 occupancy was enhanced on AMC011 and TRO.11 SOSIP-I53-50A. Furthermore, SOSIP-I53-50A trimers had elevated levels of oligomannose glycans at N88. The addition of I53-50A possibly restricts glycan processing enzyme activity at this site on gp120 close to the base of the SOSIP trimer.

To assemble the NPs, SOSIP-I53-50A and I53-50B -which was expressed in *E*. *coli* and purified from clarified lysate using affinity purification followed by SEC as published previously [45]- were mixed at an equimolar ratio and incubated overnight. For mosaic NP assembly, SOSIP-I53-50A components were mixed at equimolar ratios before adding I53-50B. NP preparations were further purified using SEC to remove unassembled components (Fig 2B). Formation of particles was confirmed by nsEM (Fig 2C), and Blue Native-PAGE analysis showed the expected megadalton-scale species consistent with NPs (S9 Fig). DLS showed that the NP preparations were monodisperse and had *R*_*h*_ of 252–292 Å (Table 1). A previous study using a targeted mass spectrometry-based selected reaction monitoring assay confirmed that this assembly method led to mosaic NP formation [46]. It was demonstrated that mixing equimolar amounts of different proteins fused to I53-50A resulted in stochastic assembly leading to a Gaussian distribution of occupancies. We conclude that all five SOSIP trimers are efficiently incorporated into well-defined and homogeneous protein NPs.

Ab binding to SOSIP, SOSIP-I53-50A and SOSIP-I53-50 NP was compared using a BLI-based assay (S10 Fig). Binding of quaternary structure-specific bNAb PGT145, CD4 binding-site bNAb VRC01, and CD4 binding-site non-NAb F105 was normalized to 2G12 binding for all constructs. PGT145 binding was generally stronger to the SOSIP-I53-50 NPs compared to the SOSIPs and SOSIP-I53-50A trimers, while VRC01 binding was generally weaker to SOSIP-I53-50 NPs compared to the SOSIP and SOSIP-I53-50A trimers. This is in line with previous observations that Ab binding to apex epitopes is improved by I53-50 display, while Ab binding to epitopes lower on the SOSIP trimer can be reduced [32]. Non-NAb F105 did not bind any of the proteins.

### Multimeric and multivalent antigen presentation improves the induction of heterologous Ab responses

**Fig 3:**
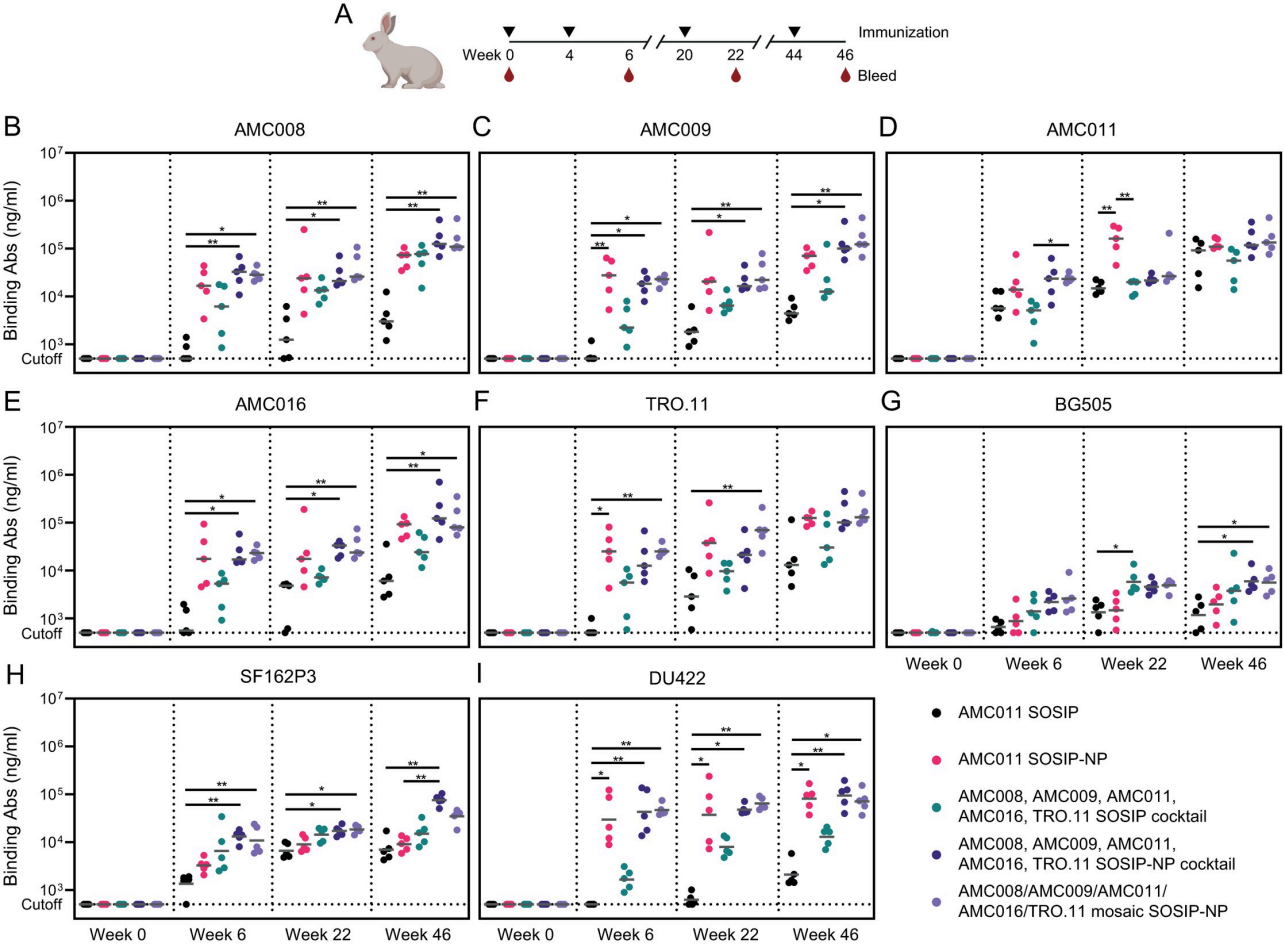
Multivalency and multimeric NP display of SOSIPs induce improved heterologous Ab binding responses. (A) Schematic overview of the immunization study. The triangles indicate immunization weeks, and the drops indicate bleeds for serum collection. (B) Ab binding measured by ELISA against AMC008 SOSIP, (C) AMC009 SOSIP, (D) AMC011 SOSIP, (E) AMC016 SOSIP, (F) TRO.11 SOSIP, (G) BG505 SOSIP, (H) SF162P3 SOSIP, and (I) DU422 SOSIP. (B-I) Sera collected at week 0, 6, 22, and 46 were analyzed. The horizontal gray bars indicate the medians. The Ab binding between groups were compared using the Kruskal-Wallis test, followed by Dunn’s post-test (*, *p* < 0.05; **, *p* < 0.01). A part of this figure was created with BioRender.

To assess the immunogenicity of the NPs, rabbits were immunized four times with 30 μg of SOSIP or 30 μg of SOSIP presented on I53-50 NPs (n = 5 per group; Fig 3A). The first two groups were immunized with AMC011 SOSIP or AMC011 SOSIP-NP to assess the effect of NP display on the immunogenicity of this clade B trimer. AMC011 SOSIP was selected for the monovalent groups for a number of reasons. First, we previously obtained a lot of experience and data using this protein [24,41,47–49], and in some studies it was found that AMC011 SOSIP induced more cross-reactive neutralizing antibody responses than other SOSIP trimers [24,41]. Second, AMC011 Env was derived from an elite neutralizer from which we isolated a bNAb against the fusion peptide, ACS202 [47,48]. Third, substantial structural information on AMC011 SOSIP was obtained, in comparison with non-stabilized WT and full-length AMC011 Env [49]. Lastly, AMC011 SOSIP (SOSIP.v8.2) has been manufactured under current good manufacturing practice conditions and is being tested in clinical studies. The third group was immunized with a cocktail of the AMC008, AMC009, AMC011, AMC016 and TRO.11 SOSIPs to assess the effect of multivalency on the breadth of the immune response. Group four was immunized with a cocktail of AMC008, AMC009, AMC011, AMC016 and TRO.11 SOSIP-NPs to assess the effect of particle display on broadening responses. Finally, group five was immunized with the mosaic NP displaying all five SOSIP constructs. Immunizations were performed at weeks 0, 4, 20, and 44 and the animals were bled at the day of each immunization and two weeks later (Fig 3A). Whole blood for peripheral blood mononuclear cell (PBMC) isolation was collected at week 45. In the first three immunizations, the immunogens were adjuvanted with Adjuplex [50–52]. After this point, this adjuvant was no longer available, therefore, the fourth immunization was performed with a squalene-based oil-in-water emulsion as the adjuvant [53,54]. This adjuvant has performed well in a number of rabbit and non-human primate (NHP) immunization studies with SOSIP trimers [17,54,55].

Ab binding to AMC008, AMC009, AMC011, AMC016, and TRO.11 SOSIPs was measured in the serum of all rabbits using ELISA at week 0 and two weeks after each immunization starting at week 6 (Fig 3B–3F). Ab binding responses were compared to a standard curve of polyclonal rabbit IgG so that a semiquantitative measure of specific IgG concentrations could be obtained. AMC011 SOSIP-NP induced improved autologous Ab responses compared to AMC011 SOSIP. This difference was most pronounced after three immunizations (median IgG titers of 162 versus 15 μg/mL, *p* = 0.0059), and confirms the benefits of NP presentation of Env trimers [18,32]. After the fourth immunization however, the amount of Abs induced by AMC011 SOSIP-NP did not increase further while the Abs induced by the SOSIP did, reducing the difference between the Ab responses induced by the two immunogens (median IgG titers of 109 versus 92 μg/mL). AMC011 SOSIP-NP induced >10-fold improved Ab responses against the other clade B proteins compared to AMC011 SOSIP. This trend was most pronounced after the fourth immunization (median IgG titers of 73 versus 3 μg/mL against AMC008 SOSIP; 70 versus 4 μg/mL against AMC009 SOSIP; 93 versus 6 μg/mL against AMC016 SOSIP; 125 versus 13 μg/mL against TRO.11 SOSIP, respectively), but the differences never reached statistical significance. Thus, the Ab responses induced by AMC011 SOSIP-NP after four immunizations were strong against all five clade B proteins, while AMC011 SOSIP induced weak responses against the heterologous clade B proteins, i.e. AMC008, AMC009, AMC016, TRO.11, showing that NP presentation augments cross-reactive Ab responses.

The SOSIP-NP cocktail induced ~5-fold stronger Ab responses compared to the SOSIP cocktail against all five autologous SOSIP proteins after two immunizations (median IgG titers of 33 versus 6 μg/mL against AMC008; 18 versus 2 μg/mL against AMC009; 24 versus 5 μg/mL against AMC011; 17 versus 5 μg/mL against AMC016; 13 versus 6 μg/mL against TRO.11, respectively). After three and four immunizations the Ab responses increased and the differences between the responses induced by the two immunogens decreased slightly. Although none of the above differences reached statistical significance, they support the supposition that NP presentation reinforces the immunogenicity of SOSIP trimers. These binding Ab data did not reveal a marked difference in the performance of the mosaic SOSIP-NP compared to SOSIP-NP cocktail.

Next, we analyzed whether cross-reactive Ab responses were induced that bind to the BG505, SF162P3 and DU422 SOSIPs, which represent heterologous sequences based on clade A, B and C viruses, respectively (Fig 3G–3I). Ab responses to BG505 SOSIP were generally weak. AMC011 SOSIP and SOSIP-NP induced similarly weak responses (median IgG titers of 1–2 μg/mL), while slightly higher responses were induced by the SOSIP cocktail, the SOSIP-NP cocktail, and the mosaic SOSIP-NP (median IgG titers of 4–6 μg/mL) after three and four immunizations. AMC011 SOSIP-NP induced minimally improved Ab responses against SF162P3 SOSIP compared to AMC011 SOSIP (median IgG titers of 3 versus 2 μg/mL after two immunizations, respectively), while the SOSIP cocktail elicited improved Ab responses compared to AMC011 SOSIP (median IgG titers of 5 versus 2 μg/mL, *p* = 0.0347). SOSIP-NP cocktail and mosaic SOSIP-NPs induced marginally higher Ab responses compared to AMC011 SOSIP-NP (median IgG titers of 13 and 8 versus 3, respectively). Between the second and fourth immunizations, the Ab concentrations increased ~5-fold, but the subtle differences between the groups remained. AMC011 SOSIP-NP induced relatively strong Ab binding responses against DU422 SOSIP after two immunizations (median IgG titer of 20 μg/mL), while AMC011 SOSIP induced Abs were below the limit of detection of our assay (IgG titers of <0.5 μg/mL). The SOSIP cocktail induced slightly improved Ab responses compared to the AMC011 SOSIP, which was most pronounced after three immunizations (median IgG titers of 7 versus 1 μg/mL). The SOSIP-NP cocktail and mosaic SOSIP-NPs induced ~20-fold improved Ab responses compared to the SOSIP cocktail after two immunizations (median IgG titers of 35 and 45 versus 2 μg/mL). Thus, while differences between the groups were subtle and often not statistically different, an overall picture emerges in which the SOSIP-NP cocktail and mosaic SOSIP-NPs performed best.

Lastly, Ab binding responses were measured against the I53-50 NP scaffold (S11 Fig). I53-50-binding Ab responses were high in all animals that received the SOSIP-I53-50 NP formulations (IgG titers ~50–100 μg/mL), but absent in animals that received the soluble SOSIP proteins. Anti-scaffold Ab responses were similar after two, three or four immunizations. The strong response against SOSIP-I53-50 NPs is consistent with a previous study in mice showing that this phenomenon is enhanced in NPs displaying HIV-1 trimers, while NPs were much less immunogenic when bearing more immunodominant antigens such as RSV F and the SARS-CoV-2 spike RBD [27].

### Clade B Env trimers elicit NAb responses to bNAb epitopes

Neutralization against the autologous viruses AMC008, AMC009, AMC011, AMC016, and TRO.11; the heterologous clade B viruses REJO and SF162P3; and the global panel viruses Ce1176_A3, 25710–2.43, BJOX002000.03.2, X1632-S2-B10, 246-F3_C10_2, CH119.10, Ce703010217_B6 and CNE55 was measured using a pseudovirus-based assay. Rabbit sera from week 6 were tested against the autologous viruses, sera from week 22 against the autologous viruses and the heterologous clade B strains, and week 46 sera were tested against all the aforementioned strains.

**Fig 4:**
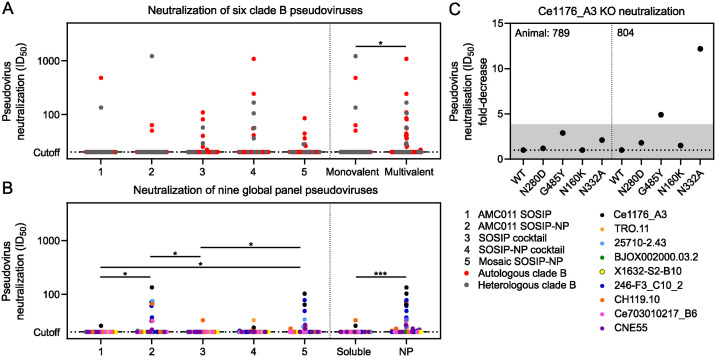
Neutralizing responses induced by Env proteins were overall low and sporadic. (A) pseudovirus neutralization of the AMC008, AMC009, AMC011, AMC016, TRO.11, REJO, and SF162P3 clade B strains by sera collected at week 46. Each dot represents an individual serum sample. Red dots show neutralization of autologous viruses, while gray dots show neutralization of heterologous viruses. (B) pseudovirus neutralization of the Ce1176_A3, 25710–2.43, BJOX002000.03.2, X1632-S2-B10, 246-F3_C10_2, CH119.10, Ce703010217_B6 and CNE55 global panel strains by sera collected at week 46. Each dot represents an individual serum sample. (A and B) The horizontal black bars indicate the medians. Groups were compared using the Kruskal-Wallis test, followed by Dunn’s post-test (*, *p* < 0.05). The monovalent and multivalent, and soluble and NP samples were compared using the Mann-Whitney U test (*, *p* < 0.05; **, *p* < 0.01; ***, *p* < 0.001). (C) Neutralization of Ce1176_A3 mutant strains. The fold-decreases compared to neutralization of WT Ce1176_A3 are indicated. Fold-decreases shaded in gray were deemed to not be high enough to confidently claim a real difference.

After two immunizations, at week 6, no neutralization was detected in any of the samples (inhibitory dilutions at which 50% neutralization is attained (ID_50_) titers of <20) (S2 Table). At week 22, after three immunizations, one rabbit in the SOSIP-NP cocktail group was able to neutralize AMC016 at an ID_50_ titer of 1,660, and neutralize REJO and SF162P3 with ID_50_ titers of 76 and 380, respectively. In two other animals, neutralizing activity against REJO and/or SF162P3 was observed, but those animals also neutralized the murine leukemia virus (MLV) negative control (S3 Table). More neutralizing activity was observed after the fourth immunization at week 46 (Fig 4 and S4 Table). AMC011 SOSIP and SOSIP-NP induced autologous neutralization in two and one animal, respectively, and induced clade B heterologous neutralizing responses against REJO in one animal of each group (ID_50_ values of 134 and 1,213, respectively). The SOSIP and SOSIP-NP cocktails induced some sporadic clade B neutralizing responses with ID_50_ titers >100. One animal in the SOSIP cocktail-immunized group neutralized AMC011 at an ID_50_ titer of 109. Two animals that received the SOSIP-NP cocktail neutralized AMC016 at ID_50_ titers of 242 and 1,078. The latter, animal 798, which already neutralized AMC016 at week 22, also neutralized REJO and SF162P3 at ID_50_ titers of 166 and 105, respectively. The mosaic SOSIP-NP-immunized animals showed some sporadic neutralization against clade B viruses with ID_50_ titers <100. Neutralization of global panel viruses was low overall and sporadic throughout all the groups. Two animals, in the AMC011 SOSIP-NP and mosaic SOSIP-NP groups, neutralized Ce1176_A3 at ID_50_ titers of 134 and 103, respectively. Overall, clade B viruses were neutralized more frequently by the groups that were immunized with all five constructs compared to the groups that only received AMC011 (Fig 4A). Global panel viruses were neutralized more frequently in the NP groups compared to the soluble antigen groups (Fig 4B).

To understand which epitopes were being targeted in the global panel neutralizing responses, we tested the sera that could neutralize Ce1176_A3 with an ID_50_ titer of >100 against four mutant Ce1176_A3 viruses: Ce1176_A3.N280D (CD4 binding site (CD4bs) resistance), Ce1176_A3.G458Y (CD4bs resistance), Ce1176_A3.N160K (V2-glycan resistance), Ce1176_A3.N332A.4 (V3-glycan resistance). Neutralization of these viruses was tested in the sera of animals 789 and 804 at week 46, and compared to the neutralization of WT Ce1176_A3 (Fig 4C). Neutralization of the mutant viruses by animal 789 was comparable to the neutralization of WT Ce1176_A3, and therefore they did not provide information on the epitope targeting for neutralization. For animal 804, we observed a 5-fold reduction in ID_50_ titer against Ce1176_A3.G458Y, and >10-fold reduction against Ce1176_A3.N332A.4, compared to the WT virus (Fig 4C). These results suggest that the neutralizing antibody responses produced by animal 804 target (an) epitope(s) at or near the CD4bs and V3-glycan bNAb epitopes.

**Fig 5:**
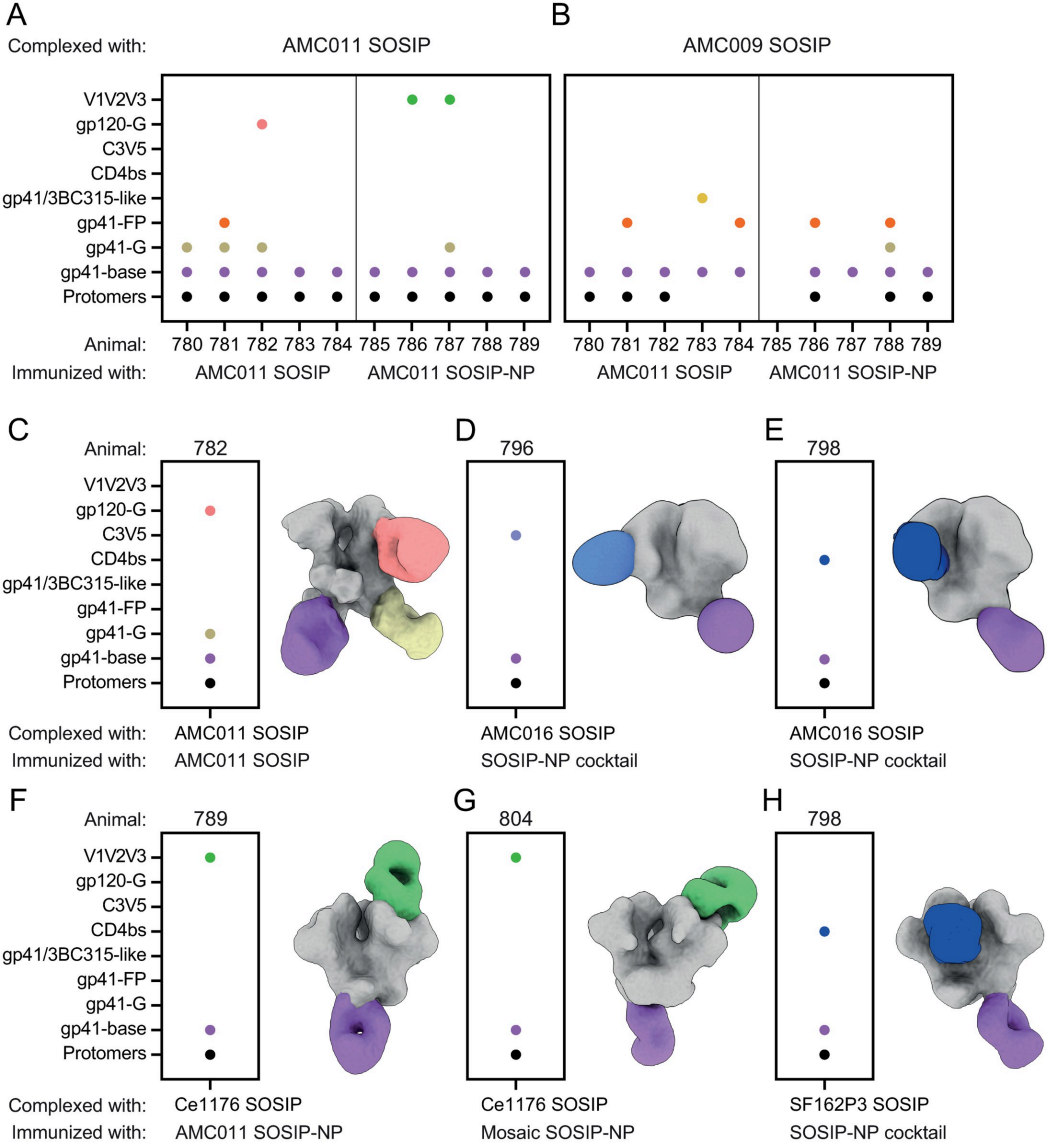
Clade B Env trimers elicit Ab responses to bNAb epitopes. (A) Fabs from animals immunized with AMC011 SOSIP and AMC011 SOSIP-NP were complexed with AMC011 SOSIP. Each dot represents an epitope that was targeted by the Ab response. (B) Fabs from animals immunized with AMC011 SOSIP and AMC011 SOSIP-NP were complexed with AMC009 SOSIP. Each dot represents an epitope that was targeted by the Ab response. (C-H) Fabs from animals that neutralized any of the tested pseudoviruses with an ID_50_ titer >100 were complexed with the corresponding SOSIP proteins. Each dot represents an epitope that was targeted by the Ab response. (C) Fabs from animal 782 were complexed with AMC011 SOSIP. (D) Fabs from animal 796 were complexed with AMC016 SOSIP. (E) Fabs from animal 798 were complexed with AMC016 SOSIP. (F) Fabs from animal 789 were complexed with Ce1176 SOSIP. (G) Fabs from animal 804 were complexed with Ce1176 SOSIP. (H) Fabs from animal 798 were complexed with SF162P3 SOSIP.

To visualize the dominant Ab specificities of the polyclonal responses, we employed electron microscopy polyclonal epitope mapping (EMPEM). Fragment antigen-binding regions (Fabs) from the week 46 sera were complexed with multiple SOSIP proteins. First, Fabs from the AMC011 SOSIP- and the AMC011 SOSIP-NP-immunized groups were complexed with AMC011 to assess the effect of NP display on the induced responses (Fig 5A). In the AMC011 SOSIP-immunized animals, four Ab responses were identified targeting epitopes at or near: one site on the gp120 subunit (gp120-glycan area (gp120-G), 1/5 rabbits), three sites on gp41 (3BC315-like, 1/5 rabbits; fusion peptide, 1/5 rabbits; gp41-G, 2/5 rabbits) and the trimer base, 5/5 rabbits, a neo-epitope created by the removal of the Env transmembrane region. Additionally, we observed antibody-induced Env subunit timer degradation, which is often caused by base targeting Abs, in 5/5 rabbits, an Ab response that causes Env trimer instability [56].

In the AMC011 SOSIP-NP-immunized animals, only two possible target sites were identified: one site overlapping with variable loops on gp120 (V1/V2/V3, 2/5 rabbits) and the trimer base (5/5 rabbits). Antibody-induced Env subunit timer degradation was also observed in 5/5 animals. I53-50 NP display of SOSIP constructs promotes the targeting of apex-proximate epitopes, as epitopes lower on the SOSIP trimer are less accessible for Ab binding. Comparing the Ab responses in AMC011 SOSIP- and AMC011 SOSIP-NP-immunized animals, it appears that Ab responses were redirected towards apex epitopes, consistent with previous findings that NP presentation can cause such redirection [18,32]. Base-targeting Ab responses were observed in all the SOSIP and SOSIP-NP immunized animals. In a previous study using I53-50 NP displaying a clade C SOSIP, base responses were also present in all animals immunized, but the Ab responses were quantitatively lower in SOSIP-NP immunized animals compared to animals immunized with the soluble SOSIP protein [18].

Second, Fabs from the AMC011 SOSIP- and the AMC011 SOSIP-NP-immunized groups were complexed with AMC009 SOSIP to assess whether the same Ab redirection would be observed in heterologous responses (Fig 5B). Three possible target sites were identified in the AMC011 SOSIP immunized animals: two on the gp41 subunit (3BC315-like, 1/5 rabbits; fusion peptide, 2/5 rabbits) and the trimer base (5/5 rabbits). In 3/5 rabbits we identified antibody-induced Env subunit timer degradation. In the AMC011 SOSIP-NP-immunized animals three target sites were identified: two gp41 sites (fusion peptide, 2/5 rabbits; gp41-G, 1/5 rabbits) and the trimer base (4/5 rabbits). In this group, antibody-induced Env subunit timer degradation was observed in 3/5 animals. Interestingly, Ab responses that were not observed against the autologous Env were observed against AMC009 in animals 783, 784, 786, and 788. These are likely subdominant responses that were not picked up in the AMC011 complexes because of low quantities. When assessing Fabs from AMC011 SOSIP- and AMC011 SOSIP-NP-immunized animals against AMC009 SOSIP, all Ab responses we could observed were directed against gp41 epitopes (Fig 5B), which is in line with the relative conservation of gp41 compared to gp120.

Third, Fabs from animals that showed neutralization with ID_50_ >100 were complexed with SOSIPs based on the viruses they neutralized. Fabs from rabbit 782, which neutralized AMC011 pseudovirus with an ID_50_ of 478, exhibited, next to a 3BC315-like response, a gp120-G-targeting response (Fig 5C). This response is similar to the N234/N276/N355 targeting response that was previously identified in an AMC011 SOSIP-immunized rabbit that showed autologous neutralization [24]. Fabs from animals 782 and 785, immunized with AMC011 SOSIP, and animal 798, immunized with the SOSIP-NP cocktail, were complexed with REJO SOSIP. Only base-targeting Ab responses and antibody-induced Env subunit timer degradation were identified in all three animals (S12 Fig). These responses are not likely to be neutralizing, suggesting that the antibodies responsible for the observed neutralizing activity were not picked up by EMPEM. Fabs from animals 796 and 798, which were both immunized with the SOSIP-NP cocktail, were complexed with AMC016 SOSIP (Fig 5D and 5E). A C3V5-directed response was observed in animal 796 and a CD4bs-directed response was observed in animal 798. Fabs from animals 789 and 804, immunized with AMC011 SOSIP-NP and mosaic SOSIP-NP, respectively, were complexed with Ce1176_A3 SOSIP (Fig 5F and 5G). A V1/V3-targeting response was observed in both animals. In animal 804 the V3-N332 site was identified as a neutralizing epitope in the KO-virus neutralization assay (Fig 4C), which is in line with the EMPEM data. Fabs from animal 798, which was immunized with the SOSIP-NP cocktail, was complexed with SF162P3 SOSIP (Fig 5H). The CD4bs was identified as an Ab target, similar to Fabs from animal 798 complexed with AMC016 SOSIP. Base-targeting responses and antibody-induced Env subunit trimer degradation were identified in all the above samples.

Overall, we conclude that NAb responses were fairly weak and sporadic. However, mapping of the neutralizing responses revealed Abs targeting multiple broadly neutralizing epitopes, demonstrating that the immunogens used here are capable of inducing responses to desired epitopes.

### A cocktail of monovalent Env trimer NPs induces superior cross-reactive B cells

To gauge cross-reactivity at the individual B cell level, we assessed the number of antigen-specific IgG^+^ B cells induced by the AMC011 SOSIP-NP, SOSIP-NP cocktail, and mosaic SOSIP-NP vaccinations using flow cytometry (S13A Fig). PBMCs collected one week after the final immunization were stained with three clade B antigens, AMC009, AMC011, and AMC016 SOSIP, as well as the heterologous DU422 SOSIP trimer. The antigens were all coupled to a shared fluorophore as well as a unique fluorophore. Only cells that were stained by the shared fluorophore and at least one of the unique fluorophores were considered. SARS-CoV-2 S was included as a negative control, and any cells cross-interacting with this protein were excluded from further analysis. Because of limitations in the number of available fluorophores and channels, we chose to include three of the five autologous probes, alongside a heterologous probe and a negative control. As the cells were stained with all probes simultaneously, we were able to determine the cross-reactivity of the antigen-specific cells using Boolean gating, which creates cell populations within multiple specified gates (S13A Fig). To further quantify the Boolean analysis, a previously described mathematical function was applied to the data, resulting in a cross-reactivity index [57].

**Fig 6:**
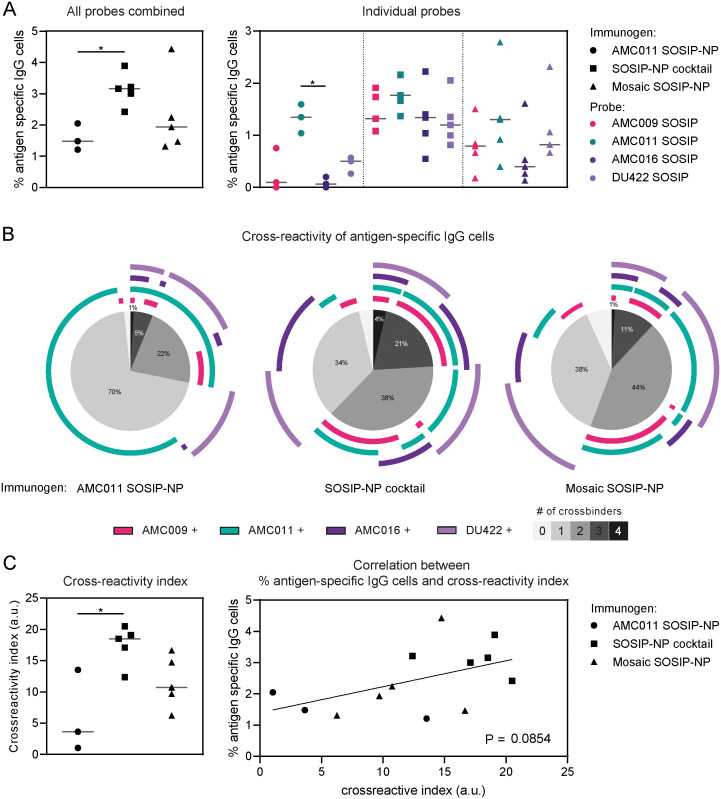
A cocktail of monovalent SOSIP-NPs displaying Env proteins induces improved cross-reactivity compared to a mosaic SOSIP-NP. PBMCs collected at week 45, after four immunizations, were stained with AMC009 SOSIP, AMC011 SOSIP, AMC016 SOSIP, DU422 SOSIP, and SARS-CoV-2 S, coupled to fluorophores, and analyzed by flow cytometry. Any cells interacting with SARS-CoV-2 S were excluded from further analysis. (A) The percentage of IgG^+^ cells interacting with the tested antigens is indicated on the left. The percentage of IgG^+^ cells interacting with each of the individual tested antigens is indicated on the right. (B) As the PBMCs were stained with all probes simultaneously, cross-reactivity within the antigen-specific cells could be determined using Boolean analysis. The pie-charts indicate the percentage of antigen-specific cells cross-interacting with the tested antigens. The different shades of gray indicate the amount of test antigens the cells interacted with. The arches around the pie-charts indicate the antigens that were bound by the cells. (C) Using a mathematical function for quantifying cross-reactivity (previously described by Larsen *et al*.) [57], a cross-reactivity index could be calculated for each vaccination group (left). On the right a correlation is shown between the percentage of antigen-specific cells and the cross-reactivity index for each animal. (A and C) Groups were compared using the Kruskal-Wallis test, followed by Dunn’s post-test (*, *p* < 0.05).

In the PBMCs of AMC011 SOSIP-NP immunized animals we observed a median of 1.5% antigen-specific IgG^+^ B cells, binding the AMC009, AMC011, AMC016, and/or DU422 SOSIP probes (Fig 6A). The antigen-specific cells induced by the SOSIP-NP cocktail were ~2-fold higher (median of 3.2% of antigen-specific IgG^+^ B cells versus 1.5%, *p* = 0.0485), while the amount of antigen-specific cells induced by the mosaic SOSIP-NP was similar compared to the AMC011 SOSIP-NP induced antigen-specific cells (median of 1.9%). AMC011 SOSIP-NP induced antigen-specific cells that mainly bound AMC011 SOSIP, while the antigen-specific cells induced by the SOSIP-NP cocktail and mosaic SOSIP-NP bound all the probes (Fig 6A).

The antigen-specific cells induced by the AMC011 SOSIP-NP almost all bound AMC011 SOSIP (Fig 6B). A few cells bound exclusively to DU422 SOSIP, which we surmise relates to its lower stability resulting in trace amounts of trimer degradation products. Of the antigen-specific cells induced by AMC011 SOSIP-NP, 70% bound only one antigen, 22% cross-interacted with two of the tested antigens, 5% interacted with three antigens, and 1% with four antigens (Table 2). The SOSIP-NP cocktail induced more cross-reactivity within the antigen-specific IgG^+^ B cell pool compared to the AMC011 SOSIP-NP. While 34% of the antigen-specific cells only bound one of the tested antigens, 38% interacted with two antigens, 21% with three antigens, and 4% with all four test antigens. In the mosaic SOSIP-NP group 38% of the antigen-specific cells bound one of the tested antigens, 44% bound two antigens, 11% interacted with three antigens, and 1% interacted with all four of the tested antigens. A cross-reactivity index was calculated for each vaccination group (Fig 6C). The cross-reactivity index of the SOSIP-NP cocktail immunized group was significantly higher compared to the AMC011 SOSIP-NP group, and a trend of improved cross-reactivity was observed in the mosaic SOSIP-NP immunized group compared to AMC011 SOSIP-NP. A clear trend of increased cross-reactivity was also observed comparing the SOSIP-NP cocktail immunized group to the mosaic SOSIP-NP immunized animals (this difference was statistically significant when the groups were directly compared with the Mann-Whitney U test, *p* = 0.0317). A trend of overall % antigen-specific B cells correlating with cross-reactivity was observed, but this was not statistically significant (Fig 6C).

**Table 2:** Cross-reactivity of antigen-specific IgG^+^ B cells.

Antigen-specific IgG^+^ B cells interacting with:	1 probe (%)	2 probes (%)	3 probes (%)	4 probes (%)
Immunogen				
AMC011 SOSIP-NP	70	22	8	1
SOSIP-NP cocktail	34	38	21	4
Mosaic SOSIP-NP	38	44	11	1

For animals 398 and 399, which were both immunized with the SOSIP-NP cocktail, more PBMCs were available, which allowed for an additional experiment in which cells were stained with all five autologous antigens. Cross-reactive cells were observed for mainly AMC008, AMC009, AMC011, and AMC016 SOSIP, while only few cells bound TRO.11 SOSIP (S13B Fig).

## Discussion

An effective HIV-1 vaccine should be able to induce a broadly reactive response to overcome HIV-1 diversity. For decades, the HIV-1 vaccine field has been working toward a vaccine that can elicit bNAbs. The design of stable and immunogenic Env constructs has brought us closer to this goal. In this study, we combined multiple structure-guided vaccine design approaches with the goal of inducing consistent cross-reactive HIV-1 neutralizing responses, using mature Env constructs. Envs were stabilized using the SOSIP.v9 design. Previous studies have demonstrated improved induction of NAbs using highly stabilized constructs [16,17]. The Env glycan shields were optimized to avoid any strain-specific glycan hole responses. Also, PNGS NxS motifs were replaced by NxT motifs where appropriate, to maximize glycan occupancy [26]. To promote a cross-reactive response, five clade B sequences were selected for a multivalent vaccine formulation: AMC008, AMC009, AMC011, AMC016, and TRO.11. The SOSIPs were presented on two-component I53-50 NPs, which have been shown to improve NAb titers in multiple studies [18,32]. All five SOSIP constructs were co-displayed on I53-50 NPs to promote interaction with cross-reactive B cells. Rabbits were immunized four times, with long intervals between immunizations.

Differences in Ab binding responses were observed after immunizations with multivalent and single antigens, and NP-displayed or soluble antigens. Multivalency improved Ab binding responses against SOSIP proteins based on heterologous clade A, B, and C strains: BG505, SF162P3, and DU422, respectively. The differences in binding Abs were 2-3-fold against BG505, 4-5-fold against SF162P3, and ~10-fold against DU422 when comparing the responses elicited by AMC011 SOSIP- and SOSIP cocktail-immunized animals at the peak differences. We did not immunize with each of the SOSIPs individually, so we cannot rule out that one of the constructs in the cocktail caused the improved responses. NP display of the SOSIP constructs improved autologous Ab binding responses up top 5-fold, mainly at the earlier time points. However, the biggest increase in Ab binding was observed against heterologous antigens. Presenting AMC011 SOSIP on I53-50 improved heterologous Ab binding responses by more than 10-fold against clade B SOSIPs AMC008, AMC009, AMC016, and TRO.11, and clade C SOSIP DU422, while the responses against the SF162P3 and BG505 SOSIPs were similar or slightly improved at certain time points. The SOSIP-NP cocktail and the mosaic SOSIP-NP induced similarly improved responses against the BG505, SF162P3 and DU422 SOSIPs compared to the SOSIP cocktail, consistent with other studies showing that SOSIP presentation on I53-50 improved Ab binding responses [18,32].

Neutralizing responses induced by the immunogens tested here were sporadic and fairly weak. We observed slightly improved neutralizing breadth induced by multivalency and NP display, but NAb titers remained low overall. Similar to the data shown here, other studies have demonstrated that improved Ab binding responses induced by SOSIP-NP immunogens do not always translate to potent NAb responses [32]. It has been demonstrated that Env constructs comprising immunogenic neutralizing epitopes near the trimer apex induce improved NAb titers when presented on NPs, while others do not [58]. Also, NP display of Env constructs has been shown to redirect Ab responses toward the trimer apex [32]. Using EMPEM, we demonstrated that in some animals immunized with AMC011 SOSIP on I53-50, Ab responses were redirected to the V1V2V3 region. Env antigens designed to elicit NAb responses to apex-proximate epitopes will likely benefit more from display on I53-50 compared to the antigens used here.

Previous multivalent immunizations with AMC008, AMC009, and AMC011 SOSIP.v5.2s in rabbits resulted in weak NAb responses against Tier-2 isolates [24]. In some of these animals, Ab responses against bNAb epitopes were identified. In another study, a neutralizing CD4bs response was identified in an AMC016 SOSIP.v4.2-immunized animal. This response could be broadened by boosting with a multivalent formulation of AMC008, AMC009, and AMC011 SOSIP immunogens [25]. Similar to these previous studies, we identified sporadic Ab responses to bNAb epitopes. Using EMPEM, we identified Ab responses directed to sites at or near variable loops on gp120, the CD4bs, and the fusion peptide, amongst others. Interestingly, we identified a CD4bs response in an animal immunized with the SOSIP-NP cocktail, which includes AMC016 SOSIP. As a CD4bs response was induced with a fully glycosylated AMC016 SOSIP before [25], AMC016 SOSIP might be a valuable immunogen for inducing and/or boosting CD4bs-directed responses.

Mosaic NP immunogens displaying influenza virus hemagglutinin [37], hepatitis C virus glycoproteins E1 and E2 [36], and diverse sarbecovirus S proteins [59], induced slightly broader and more potent neutralizing responses compared to cocktails of monovalent NPs displaying the same antigens. The lack of consistent neutralizing responses here makes it difficult to assess the effectiveness of the mosaic NP HIV-1 SOSIP display. However, we were able to assess the antigen-specific IgG^+^ B cell responses induced by the different NP immunogens. Cross-reactivity within the antigen-specific B cell pools was analyzed by flow cytometry after AMC011 SOSIP-NP, SOSIP-NP cocktail, and mosaic SOSIP-NP immunizations. Cross-reactivity of B cells induced by AMC011 SOSIP-NP was overall low compared to the multivalent vaccines, with most of the cells only binding AMC011 SOSIP. As expected, the SOSIP-NP cocktail and the mosaic SOSIP-NP induced more cross-reactive B cells compared to AMC011 SOSIP. Interestingly, the SOSIP-NP cocktail induced more cross-reactivity compared to the mosaic SOSIP-NP. We hypothesized that the mosaic immunogen would preferentially interact with, and activate, B cells recognizing conserved epitopes. The observation that the mosaic NP induced less cross-reactivity compared to the cocktail of monovalent NPs, suggests a conserved epitope was either absent or had very low affinity for the cognate B cell receptors. Presumably, B cells interacting with the monovalent NPs were stimulated more efficiently compared to B cells interacting with the mosaic NPs, because they benefited from the enhanced avidity as a consequence of NP presentation.

It is clear that the AMC008, AMC009, AMC011, AMC016, and TRO.11 mature Env constructs used here, even with all the design approaches applied, are not able to induce consistent cross-reactive neutralizing responses. The acquired data indicate that cross-reactive neutralizing responses could not be elicited, because B cell receptors were unable to recognize an immunogenic, conserved, neutralizing epitope on these Env constructs. Our results might present the ‘last nail in the coffin’ for HIV-1 vaccine approaches aimed at inducing bNAbs that do not consider germline-targeting and/or lineage-based sequential vaccination strategies, but are solely based on unmodified (but stabilized) Env trimers. Germline-targeting immunogens are designed to elicit responses against specific neutralizing epitopes [43,60,61]. It has been shown that germline-targeting immunogens are able to activate bNAb precursor germline B cells, which could be matured using tailored booster immunogens [62]. It would be interesting to explore mosaic and mixed NPs, as described in this study, in the context of germline targeting immunogens. On germline-targeting immunogens, glycan holes are often deliberately created around epitopes of interest. The booster immunogens that follow these germline-targeting immunogens should have more dense glycan shields as that is what the Abs will need to protect against. As we have seen that it is possible to induce responses to bNAb epitopes with the densely glycosylated immunogens described in this study, we believe that these constructs could be useful boosting immunogens.

There were some limitations to our study. First, the I53-50 NP scaffold induced potent Ab responses. In a previous study, it was observed that HIV-1 Env is immunosubdominant compared to the I53-50 NP scaffold [27]. Using a NP scaffold that is less immunogenic might have induced improved Env directed responses. Similarly, the immunogens induced trimer base responses which can be distracting for the immune system. Reducing the immunogenicity of the trimer base could improve responses to more relevant epitopes on the Env trimers. Second, we only sorted memory B cells, while serum antibodies derive from plasmablasts and plasma cells. This might have led to discrepancies between Ab binding measured by ELISA and antigen-specific B cells measured by flow cytometry. Third, there are well-known differences in the B cell receptor repertoire of rabbits compared to NHPs and humans [63], we cannot formally exclude that, if we had used a different experimental model, the outcomes of our experiments would have been different.

## Methods

### Ethics statement

Immunizations were performed under permits with approval number C0031-20. Immunization procedures complied with all relevant ethical regulations and protocols of the Covance Institutional Animal Care and Use Committee.

### Construct design

The Env sequences of AMC008 (PP544452) [15], AMC009 (PP544519) [24], AMC011 (PP544654) [48], AMC016 [41], and TRO.11 (AY835445) [64] were used for the immunization study. To make the SOSIP.v9 constructs, the following mutations were included in the Env sequences: 47D, 49E, 49C, 64K, 65K, 66R, 73C, 106E, 165L, 271I, 288L, 304V, 315Q, 316W, 319Y, 363Q, 429R, 432Q, 500R, 501C, R6, 519S, 535M, 543Q, 555C, 559P, 561C, 568D, 570H, 585H, 605C and ΔMPER. For glycan optimization the following mutations were included in the SOSIP sequences: AMC008 189NxT, 236T, 613T; AMC009 189NxT, 411NxT, 613T; AMC011 188NxT, 613T; AMC016 188NxT, 613T; TRO.11 134NxS, 141T, 199T, 611NxT. A MDRAKLLLLLLLLLLPQAQ artificial signal peptide was used in all the constructs. DNA constructs were ordered (Integrated DNA Technologies) and cloned by Gibson assembly into a PstI and BamHI digested pPPI4 backbone containing no tag, 6xHistidine tag, AVI tag or I53-50A.

### SOSIP production

For SOSIP production, an expression plasmid was transiently transfected into HEK 293F cells (cultured in Freestyle medium (Life Technologies)) together with a plasmid containing furin for protein cleavage at a 2:1 ratio. DNA (312.5 μg/L cells) was mixed with PEImax (937.2 μg/L cells) in OptiMEM (Gibco) and added to the cells at a density of 0.8–1.2 million cells/mL. Cell cultures were left at 37°C with 8% CO_2_ shaking at 125 rpm for 6 days. Six days post transfection, cell cultures were centrifuged at 4000 rpm for 30 min and supernatants were filtered through a 0.22 μm Steritop filter (Merck Millipore). Supernatants were subjected to a PGT145 (AMC008 SOSIP.v9, AMC009 SOSIP.v9, TRO.11 SOSIP.v9, BG505.v4.1, DU422 SOSIP.v4.1) or PGT151 (AMC011 SOSIP.v9, AMC016 SOSIP.v9, SF162P3 SOSIP.v9) affinity column. Proteins were eluted from the column and concentrated using 100 kDa cutoff Vivaspin filters (GE healthcare) and buffer exchanged to TN75 (20 mM TrisHCl pH 8.0, 75 mM NaCl). Proteins were further purified using a Superose 6 increase 10/300 GL column (GE healthcare) in TN75 or assembly buffer (25 mM TrisHCl pH 8.0, 500 mM NaCl, 5% glycerol) for SOSIP-I53-50A constructs. Appropriate size fractions were collected and pooled. Proteins were concentrated using 100 kDa cutoff Vivaspin filters and stored at -80°C.

### I53-50B.4PT1 production

I53-50B.4PT1 was produced in Lemo21(DE3) (NEB) cultured in LB medium (10 g Tryptone, 5 g Yeast Extract, 10 g NaCl) in 2 L baffled shake flasks or a 10 L BioFlo 320 Fermenter (Eppendorf). The cells were cultivated at 37°C until reaching an OD600 ∼0.8, followed by induction with 1 mM IPTG. Subsequently, the cells were grown for approximately 16 hours at 18°C. Harvested cells were lysed using a Microfluidics M110P at 18,000 psi in 50 mM Tris, 500 mM NaCl, 30 mM imidazole, 1 mM PMSF, and 0.75% CHAPS. The lysates underwent centrifugation at 24,000 g for 30 min and were then applied to a 2.6 × 10 cm Ni Sepharose 6 FF column (Cytiva) for purification via IMAC on an AKTA Avant150 FPLC system (Cytiva). The protein of interest was eluted using a linear gradient of 30 mM to 500 mM imidazole in 50 mM Tris pH 8.0, 500 mM NaCl, and 0.75% CHAPS buffer. Collected fractions were pooled, concentrated using 10 kDa cutoff centrifugal filters (Millipore), sterile filtered (0.22 μm), and applied to either a Superdex 200 Increase 10/300 or HiLoad S200 pg GL SEC column (Cytiva) using 50 mM Tris pH 8.0, 500 mM NaCl, and 0.75% CHAPS buffer. I53-50B.4PT1 eluted at ∼0.45 CV. Post-sizing, bacterial-derived components were examined to ensure low levels of endotoxin before utilization in nanoparticle assembly.

### I53-50-SOSIP nanoparticle assembly

After size exclusion chromatography (SEC) of SOSIP-I53-50A, appropriate fractions were pooled and I53-50B was added in equimolar amounts. For mosaic particle assembly, SOSIP-I53-50A components were mixed at equimolar amounts before adding I53-50B. Particles were assembled overnight at 4°C and applied to a Superose 6 increase 10/300 GL column in the assembly buffer (25 mM TrisHCl pH 8.0, 500 mM NaCl, 5% glycerol) to remove unassembled components. Appropriate fractions were collected and concentrated using a 10 kDa cutoff vivaspin filter and buffer exchanged to PBS 250mM sucrose using Slide-A-Lyzer MINI dialysis device (20 kDa molecular weight cutoff; ThermoFisher Scientific). Particles were stored at -80°C.

### Ni-NTA ELISA

For SOSIP quality control, His-tagged SOSIPs were coated on Ni-NTA plates at 1 μg/mL in Tris buffered saline (TBS) for 2 h at room temperature. Plates were washed twice with TBS. Primary antibodies in TBS 2% milk at 1 μg/mL were added to the top row of the plate and serially diluted with 3x dilution steps. After 2 h of incubation, plates were washed three times with TBS. HRP-labeled goat-anti-human antibody in TBS 2% milk was added to the plates and incubated for 1 h. Plates were washed five times with TBS 0.05% Tween-20 and once with TBS. Developing solution (0.1 M NaAc + 0.1 M citric acid, 1% TMB) was added to the wells for 30 seconds after which the reaction was stopped with 0.8M H_2_SO_4_. OD450 was measured in a plate reader.

### GNL ELISA

For testing the SOSIP proteins against bNAb precursors, high-binding plates were coated with 50 μg/mL Galanthus Nivalis Lectin (GNL) in 0.1 M NaHCO_3_ overnight at room temperature. Plates were washed twice with Tris buffered saline (TBS) and plates were blocked with Casein for 30 min. After blocking, plates were washed three times with TBS and SOSIP proteins were added at 1 μg/mL in TBS with 10% fetal calf serum (FCS). After 2 h, plates were washed twice with TBS and primary Ab solutions were added to the top row (5 μg/mL for mature bNAbs and 50 μg/mL for bNAb precursors in Casein). Abs were serially diluted in steps of three times. After 2 h, plates were washed three times with TBS and the secondary goat-anti-human Ab was added (1:3000 in Casein). After 1 h, plates were washed five times with TBS containing 0.05% Tween-20. Developing solution (0.1 M NaAc + 0.1 M citric acid, 1% TMB) was added to the plates for 5 minutes after which the reaction was stopped with 0.8M H_2_SO_4_. OD450 was measured in a plate reader.

### Dynamic light scattering (DLS)

DLS was used to assess the hydrodynamic radius (Rh) and polydispersity of the SOSIP proteins. The proteins were diluted to 0.025 μg/mL in PBS and loaded into a Dynapro Nanostar instrument (Wyatt Technology Corporation). Rh and polydispersity values were measured with ten acquisitions of 5 s each at 25°C and analyzed using the manufacturer’s software (Dynamics, Wyatt Technology Corporation).

### Sypro orange differential scanning fluorimetry (DSF)

Proteins were diluted in PBS to a concentration of 0.2 mg/mL in 0.1 mL polypropylene PCR tube strips (Axygen) in a total volume of 25 μL. Sypro orange protein stain (Invitrogen) was added 1:200. Samples were placed in a Rotor-Gene Q (Qiagen) and a high resolution melt (HRM) run from 25°C to 90°C was done with 4 second steps of 1°C each. Melt curves were analyzed with the Rotor-Gene Q Series Software.

### Negative stain electron microscopy (NSEM)

SOSIPs were incubated with Adjuplex and added to carbon-covered 400 mesh copper grids and stained with 2% uranyl formate. SOSIP-I53-50 NPs were directly added to carbon-covered 400 mesh copper grids and stained with 2% uranyl formate. Micrographs were imaged on a Tecnai F12 Spirit microscope with a 4k FEI Eagle CCD. Leginon and Appion were used to collect and process micrographs [65].

### SDS-PAGE and Blue native PAGE analysis

4–12% Tris-Glycine gels (Invitrogen) were loaded with 2 μg of SOSIP, SOSIP-I53-50S, or SOSIP I53-50 NP protein, mixed with loading dye with, or without, dithiothreitol (DTT), which were boiled for 10 min. Gels were run at 120V for approximately 1.5 h. The SDS-PAGE gels were stained overnight using PageBlue protein staining solution (Thermo Scientific). 3–12% Bis-Tris NuPAGE gels (Invitrogen) were loaded with 2 μg of SOSIP, SOSIP-I53-50S, or SOSIP I53-50 NP protein, mixed with loading dye and run at 200V for approximately 1.5 h. Blue native PAGE gels were stained overnight using the Colloidal blue staining kit (Invitrogen).

### Biolayer Interferometry (BLI) assay

SOSIP, SOSIP-I53-50A and SOSIP-I53-50 NP were diluted to 100 nM, 100 nM and 5nM, respectively, in BLI running buffer (PBS/0.1% bovine serum albumin/0.02% Tween20). Antibody binding was assessed using a ForteBio Octet K2. The assays were performed at 30°C and with agitation set at 1000 rpm. Antibody was loaded on protein A sensors (ForteBio) at 5 μg/mL in running buffer until a binding threshold of 1 nm was reached. Association and dissociation were measured for 300 seconds.

### Site-specific N-linked glycan analysis

Three aliquots of each sample were denatured for 1h in 50 mM Tris/HCl, pH 8.0 containing 6 M of urea and 5 mM dithiothreitol (DTT). Next, Env proteins were reduced and alkylated by adding 20 mM iodoacetamide (IAA) and incubated for 1h in the dark, followed by a 1 h incubation with 20 mM DTT to eliminate residual IAA. The alkylated Env proteins were buffer-exchanged into 50 mM Tris/HCl, pH 8.0 using Vivaspin columns (10 kDa) and digested separately overnight using trypsin, chymotrypsin or alpha lytic protease (Mass Spectrometry Grade, Promega) at a ratio of 1:16 (w/w). The next day, the peptides were dried and extracted using Oasis HLB 96 well plate (Waters). The peptides were dried again, re-suspended in 0.1% formic acid and analyzed by nanoLC-ESI MS with an Ultimate 3000 HPLC (Thermo Fisher Scientific) system coupled to an Orbitrap Eclipse mass spectrometer (Thermo Fisher Scientific) using stepped higher energy collision-induced dissociation (HCD) fragmentation. Peptides were separated using an EasySpray PepMap RSLC C18 column (75 μm × 75 cm). A trapping column (PepMap 100 C18 3μM 75μM x 2cm) was used in line with the LC prior to separation with the analytical column. The LC conditions were as follows: 280-minute linear gradient consisting of 5–40% acetonitrile in 0.1% formic acid over 255 minutes followed by 20 minutes of alternating 95% acetonitrile in 0.1% formic acid and 2.5% acetonitrile in 0.1% formic acid, used to ensure all the sample had eluted from the column. The flow rate was set to 300 nL/min. The spray voltage was set to 2.5 kV and the temperature of the heated capillary was set to 55°C. The ion transfer tube temperature was set to 275°C. The scan range was 375−1500 m/z. Stepped HCD collision energy was set to 15, 25 and 45% and the MS2 for each energy was combined. Precursor and fragment detection were performed using an Orbitrap at a resolution MS1 = 120,000, MS2 = 30,000. The AGC target for MS1 was set to standard and injection time set to auto which involves the system setting the two parameters to maximize sensitivity while maintaining cycle time.

Glycopeptide fragmentation data were extracted from the raw file using Byos (Version 4.6 Protein Metrics Inc.). The glycopeptide fragmentation data were evaluated manually for each glycopeptide; the peptide was scored as true-positive when the correct b and y fragment ions were observed along with oxonium ions corresponding to the glycan identified. The MS data was searched using the Protein Metrics 305 N-glycan library with sulfated glycans added manually. The relative amounts of each glycan at each site as well as the unoccupied proportion were determined by comparing the extracted chromatographic areas for different glycotypes with an identical peptide sequence. All charge states for a single glycopeptide were summed. The precursor mass tolerance was set at 4 ppm and 10 ppm for fragments. A 1% false discovery rate (FDR) was applied. The relative amounts of each glycan at each site as well as the unoccupied proportion were determined by comparing the extracted ion chromatographic areas for different glycopeptides with an identical peptide sequence. Glycans were categorized according to the composition detected.

HexNAc(2)Hex(10) was defined as M9Glc, HexNAc(2)Hex(9−3) was classified as M9 to M3. Any of these structures containing a fucose were categorized as FM (fucosylated mannose). HexNAc(3)Hex(5−6)X was classified as Hybrid with HexNAc(3)Fuc(1)X classified as Fhybrid. Both of these categories were classified as high mannose. Complex-type glycans were classified according to the number of processed antenna and fucosylation. If all of the following compositions have a fucose they are assigned into the FA categories. HexNAc(3)Hex(3–4)X is assigned as A1, HexNAc(4)X is A2/A1B, HexNAc(5)X is A3/A2B, and HexNAc(6)X is A4/A3B. As this fragmentation method does not provide linkage information compositional isomers are grouped, so for example a triantennary glycan contains HexNAc 5 but so does a biantennary glycans with a bisect. Core glycans refer to truncated structures smaller than M3. M9Glc- M4 were classified as oligomannose-type glycans. Glycans containing at least one sialic acid or fucose were categorized as NeuAc and fucosylated respectively.

### Rabbit immunization study

Female New Zealand White rabbits aged ~6 months of 2.5–3 kg from multiple litters were used in this study. Animals were sourced and housed at Covance Research Products, Inc. (Denver, PA, USA). Five animals per group were immunized with 30 μg of soluble SOSIP or 30 μg SOSIP displayed on I53-50 NPs. Group 1, AMC011 SOSIP; Group 2, AMC011 SOSIP-I53-50 NP; Group 3, AMC008 SOSIP, AMC009 SOSIP, AMC011 SOSIP, AMC016 SOSIP and TRO.11 SOSIP cocktail; Group 4, AMC008 SOSIP-I53-50 NP, AMC009 SOSIP-I53-50 NP, AMC011 SOSIP-I53-50 NP, AMC016 SOSIP-I53-50 NP and TRO.11 SOSIP-I53-50 NP cocktail; Group 5, AMC008 SOSIP-, AMC009 SOSIP-, AMC011 SOSIP-, AMC016 SOSIP- and TRO.11 SOSIP- mosaic I53-50 NP. Animals were immunized at weeks 0, 4, and 20 with proteins adjuvanted with Adjuplex via the intramuscular route (one 0.5 mL injection in each quadricep). At week 44, animals were again immunized, but the proteins were adjuvanted in squalene emulsion, because Adjuplex was no longer available. Blood was drawn for serum collection at weeks 0, 2, 4, 6, 8, 10, 12, 16, 20, 22, 26, 46 and whole blood for peripheral blood mononuclear cells isolation was collected at weeks 21 and 45.

### Serum ELISA

Columns G and H of half area high binding plates (Greiner Bio-one) were coated with goat-anti-rabbit anti-Fc (Jackson Immunoresearch) at 1:3,000 in TBS and the rest of the plate was coated with 20 μg/mL Galanthus Nivalis Lectin in 100 mM NaHCO_3_ at 4°C overnight. Plates were washed twice with TBS. Plates were blocked with Casein for 1 h and washed three times with TBS. SOSIP 0.25 μg/mL (AMC008 SOSIP.v9, AMC009 SOSIP.v9, AMC011 SOSIP.v9, AMC016 SOSIP.v9, TRO.11 SOSIP.v9, BG505 SOSIP.v4.1, DU422 SOSIP.v4.1, SF162P3 SOSIP.v9, or I53-50 cage) in Casein was added to the lectin coated wells while the goat-anti-rabbit anti-Fc coated wells were kept in Casein. After 1 h at room temperature, plates were washed three times with TBS. Serum samples were diluted in casein 1:1,000, 1:10,000, and 100,000, and added to the SOSIP coated wells in triplo. At the same time, rabbit IgG mix (Bio-rad) was added to the top wells of columns G and H at 2 μg/mL and serially diluted with 5x dilution steps. Plates were incubated for 3 h and washed three times with TBS. Goat-anti-rabbit-HRP in casein was added to the entire plate and incubated for 1 h. Plates were washed five times with TBS 0.05% Tween-20 and once with TBS. Developing solution (0.1 M NaAc + 0.1 M citric acid, 1% TMB) was added to the wells, after which the reaction was stopped with 0.8M H_2_SO_4_. OD450 was measured in a plate reader. Binding antibody concentrations in the sera were calculated using the rabbit IgG standard curve on each individual plate.

### Flow cytometry

Flow cytometry analysis of Env-specific B cells coupled to fluorescent probes were prepared using biotinylated Env proteins as previously described [66]. Briefly, Biotinylated proteins were multimerized with fluorescently-labeled streptavidin at 4°C for 1h at a 2:1 protein to streptavidin molar ratio (BB515, BD Biosciences; AF647, Biolegend; BUV615, Biolegend; PE-Cy7, BD Biosciences; BV421, Biolegend; BUV615, BD Biosciences). 50uM biotin (Genecopoiea) was added to saturate unbound streptavidin conjugates for 15mins. Then, frozen rabbit PBMCs were thawed and resuspended in RPMI-1640 (ThermoFischer). Cells were stained with fluorescent probes for 1h at 4°C and washed twice with FACS buffer (2% fetal calf serum and 1mM EDTA in PBS). Cells were stained again with a live/dead stain (Fixable viability eF780, eBiosciences, 1:1000) and mouse anti-rabbit IgG PE (Southern Biotech, 4090–09, 1:1000) for 30 mins at 4°C. Stained samples were washed twice in FACS buffer and acquired on the BD LSRFortessa for cell analysis. Analysis was performed using FlowJo software (BD Biosciences) and cross-reactivity was analyzed using Simplified Presentation of Incredibly Complex Evaluations data software version 6 (SPICE6) (SPICE Help—Index (niaid.github.io)).

### Pseudovirus neutralization assay

Serum neutralization was measured in a TZM-bl cell luciferase reporter gene neutralization assay [67]. In short, sera were diluted 1:20 and serially diluted with 3-fold dilution steps and mixed with pseudovirus on the TZM-bl cells. After three days, cells were lysed and luciferase activity was measured. Midpoint neutralization titers (ID50-values) were determined as the serum dilution at which infectivity was inhibited by 50%. Neutralization against MLV, AMC008, AMC009, AMC011, AMC016, TRO.11, REJO, SF162P3 was measured at Amsterdam UMC in Amsterdam, The Netherlands. Neutralization against Ce1176_A3, 25710–2.43, BJOX002000.03.2, X1632-S2-B10, 246-F3_C10_2, CH119.10, Ce703010217_B6, CNE55, Ce1176_A3.N280D, Ce1176_A3.G458Y, Ce1176_A3.N160K and Ce1176_A3.N332A.4 was measured at Duke University Medical Centre in Durham, NC, USA.

### EMPEM

Serum and sample preparation for obtaining polyclonal Fabs for electron microscopy were previously detailed [68]. In summary, IgG was extracted from 0.5 mL rabbit sera collected at week 46 using Protein G (Cytiva). Papain (Sigma Aldrich) was employed to enzymatically digest IgG into Fabs. An overnight incubation with 15 μg of SOSIP and 1 mg of Fab mixture (containing Fc and residual papain) was conducted, followed by purification the next day using a Superdex 200 Increase 10/300 GL gel filtration column (Cytiva). The purified complexes were concentrated and then diluted to a final concentration of 0.03 mg/mL. Subsequently, the diluted samples were applied to glow-discharged carbon-coated copper mesh grids, and staining was carried out with 2% (w/v) uranyl formate. Electron microscopy images were acquired using an FEI Tecnai Spirit T12 equipped with an FEI Eagle 4k x 4k CCD camera (120 keV, 2.06 Å/pixel) or an FEI Tecnai TF20 equipped with a Tietz F416 CMOS camera (200 keV, 1.77 Å/pixel). The images were processed using Relion 3.0 [69], following the standard 2D and 3D classification procedures. UCSF Chimera was utilized to generate the composite maps [70].

## Supporting information

S1 Fig Glycan shield predictions, related to Fig 1. Predictions of the glycan shields of all five SOSIP proteins made using the Los Alamos glycan mapping tool. On the left are the glycan predictions of the original sequences. On the right are the glycan predictions after glycan optimization. The red arrows indicate predicted glycan holes.
(EPS)

S2 Fig SOSIP quality controls, related to Fig 1. (A) Size exclusion chromatogram of the five SOSIP constructs. The collected fractions are indicated in yellow. The graph is an overlay of multiple runs. (B) SDS PAGE gel showing the five SOSIP proteins under reducing conditions. (C) SDS PAGE gel showing the SOSIP proteins under non-reducing conditions. (D) Blue Native PAGE gel showing the five SOSIP proteins.
(EPS)

S3 Fig Melt curves for SOSIPv.5.2 and SOSIPv.9 proteins, related to Table 1. Melting temperatures obtained using a sypro orange differential scanning fluorimetry assay.
(EPS)

S4 Fig ELISA of SOSIP.v9s, related to Fig 1. ELISA curves of 2G12, PG9, PG16, PGT145, b12, VRC01, PGT151, 35022, F105 binding to all five SOSIP proteins measured by ELISA. Below is a heat map representing areas under the curve normalized to 2G12 binding.
(EPS)

S5 Fig ELISA of SOSIP.v9s with bNAb precursors, related to Fig 1. ELISA curves of 2G12, PGT151, VRC01, germline (gl)-VRC01, 12A12, gl-12A12, gl-3BNC60, PG9, and gl-PG9 binding to all five SOSIP proteins and BG505 SOSIP.v8.1 and BG505 SOSIP.v8.1 GT1.1 measured by ELISA.
(EPS)

S6 Fig Glycan occupancy on SOSIP proteins, related to Fig 1. Glycan occupancy obtained using nanoLC-ESI MS. (A-E) The percentage of glycan occupancy is indicated by bars. Green indicates high mannose glycans, pink indicates complex glycans, and gray bars indicate unoccupied motives. Positions that could not be measured were left blank. (A) AMC008 SOSIP. (B) AMC009 SOSIP. (C) AMC011 SOSIP. (D) AMC016 SOSIP. (E) TRO.11 SOSIP.
(EPS)

S7 Fig SOSIP-I53-50 NP SDS-PAGE gels, related to Fig 2. (A) SDS PAGE gel showing the six SOSIP-I53-50 NPs under reducing conditions. (B) SDS PAGE gel showing the SOSIP-I53-50 NPs under non-reducing conditions.
(EPS)

S8 Fig Glycan occupancy on SOSIP-I53-50A proteins, related to Fig 2. Glycan occupancy obtained using nanoLC-ESI MS. (A-E) The percentage of glycan occupancy is indicated. Green indicates high mannose glycan, pink indicates complex glycans, and gray bars indicate unoccupied motives. Positions that could not be measured were left blank. (A) AMC008 SOSIP-I53-50A. (B) AMC009 SOSIP-I53-50A. (C) AMC011 SOSIP-I53-50A. (D) AMC016 SOSIP-I53-50A. (E) TRO.11 SOSIP-I53-50A.
(EPS)

S9 Fig SOSIP-I53-50 NP Blue Native PAGE gel, related to Fig 2. Blue Native PAGE gel showing the six SOSIP-I53-50 NPs.
(EPS)

S10 Fig Monoclonal Ab binding to SOSIP, SOSIP-I53-50A and SOSIP-I53-50 NPs, related to Figs 1 and 2. Binding of 2G12, PGT145, VRC01, and F105, to the SOSIPs, SOSIP-I53-50As, and SOSIP-I53-50 NPs, measured using a BLI-based assay. Binding is represented as areas under the curve normalized for 2G12 binding.
(EPS)

S11 Fig Ab binding to I53-50 scaffold, related to Fig 3. Ab binding measured by ELISA against I53-50 cage. Sera collected at week 0, 6, 22, and 46 were analyzed. The horizontal gray bars indicate the medians. The Ab binding between groups were compared using the Kruskal-Wallis test, followed by Dunn’s post-test (*, *p* < 0.05).
(EPS)

S12 Fig Fabs of REJO neutralizers complexed with REJO SOSIP. Fabs from animals that neutralized the REJO pseudoviruses with an ID_50_ titer >100 were complexed with the REJO SOSIP protein. Each dot represents an epitope that was targeted by the Ab response. Fabs from animals 782, 785, and 798 were complexed.
(EPS)

S13 Fig Antigen-specific IgG^+^ cell responses, related to Fig 6. (A) Representative gating strategy for the identification of AMC009 SOSIP, AMC011 SOSIP, AMC016 SOSIP, and TRO.11 SOSIP-specific cells. (B) Cross-reactivity within the antigen-specific cells determined by using Boolean analysis. The pie-charts indicate the percentage of antigen-specific cells cross-interacting with the tested antigens. The different shades of gray indicate the amount of test antigens the cells interacted with. The arches around the pie-charts indicate the antigens that were bound by the cells.
(EPS)

S1 Table Sequence identity matrix of selected Env sequences compared to BG505 and ZM197M Env. (TIFF)

S2 Table ID_50_ neutralization titers at week 6, related to Fig 4. (TIFF)

S3 Table ID_50_ neutralization titers at week 22, related to Fig 4. (TIFF)

S4 Table ID_50_ neutralization titers at week 46, related to Fig 4. (TIFF)

S1 Raw Data Data that underlies this paper. (XLSX)
