# Supplementary figures and images for: Mosaic and mixed HIV-1 glycoprotein nanoparticles elicit antibody responses to broadly neutralizing epitopes
Source: PLoS Pathog. 2024 Oct 3;20(10):e1012558. doi: 10.1371/journal.ppat.1012558 (PMC11449375; doi:10.1371/journal.ppat.1012558)

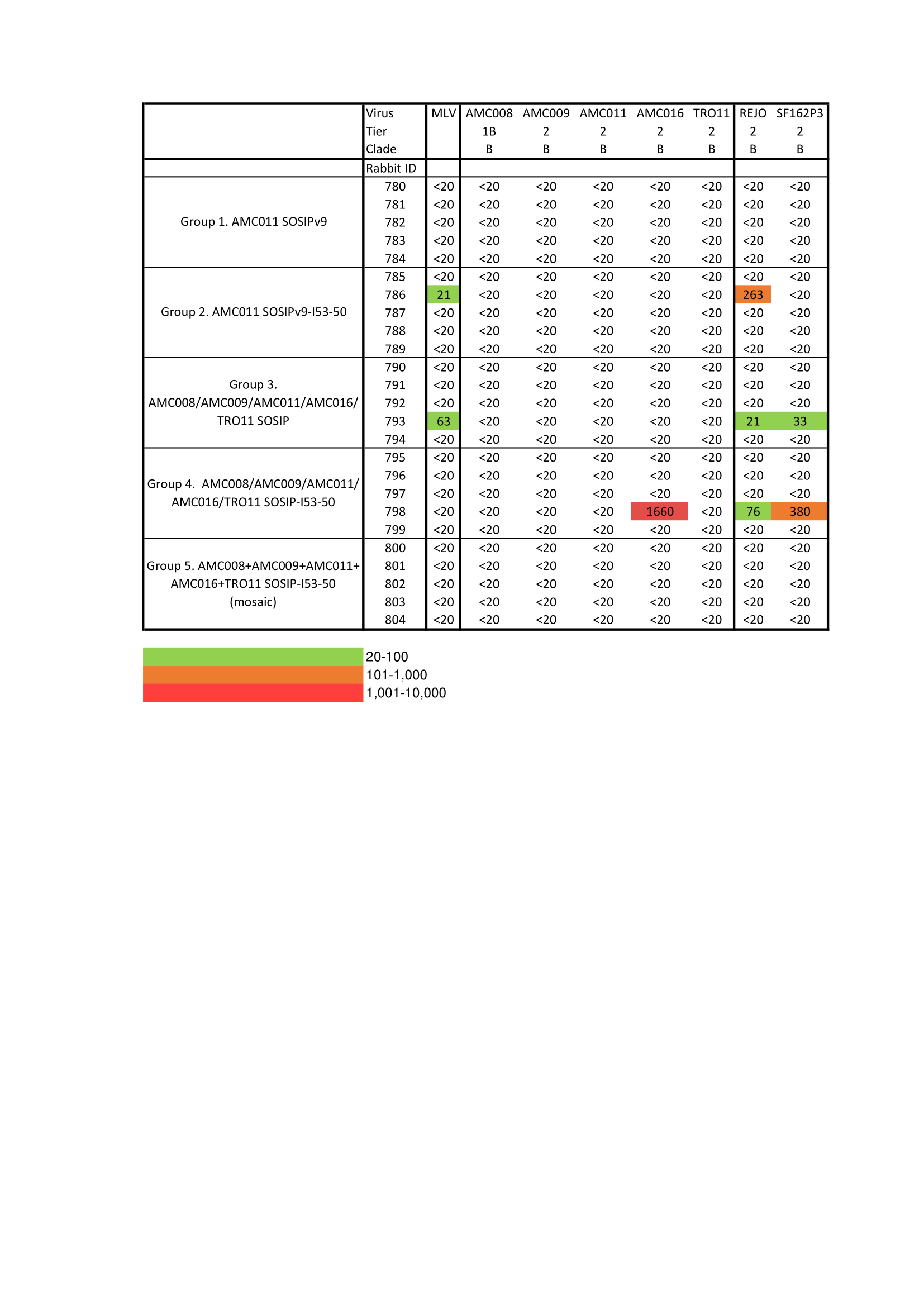

Supplement: S3 Table — (TIFF) [file ppat.1012558.s016.tiff]

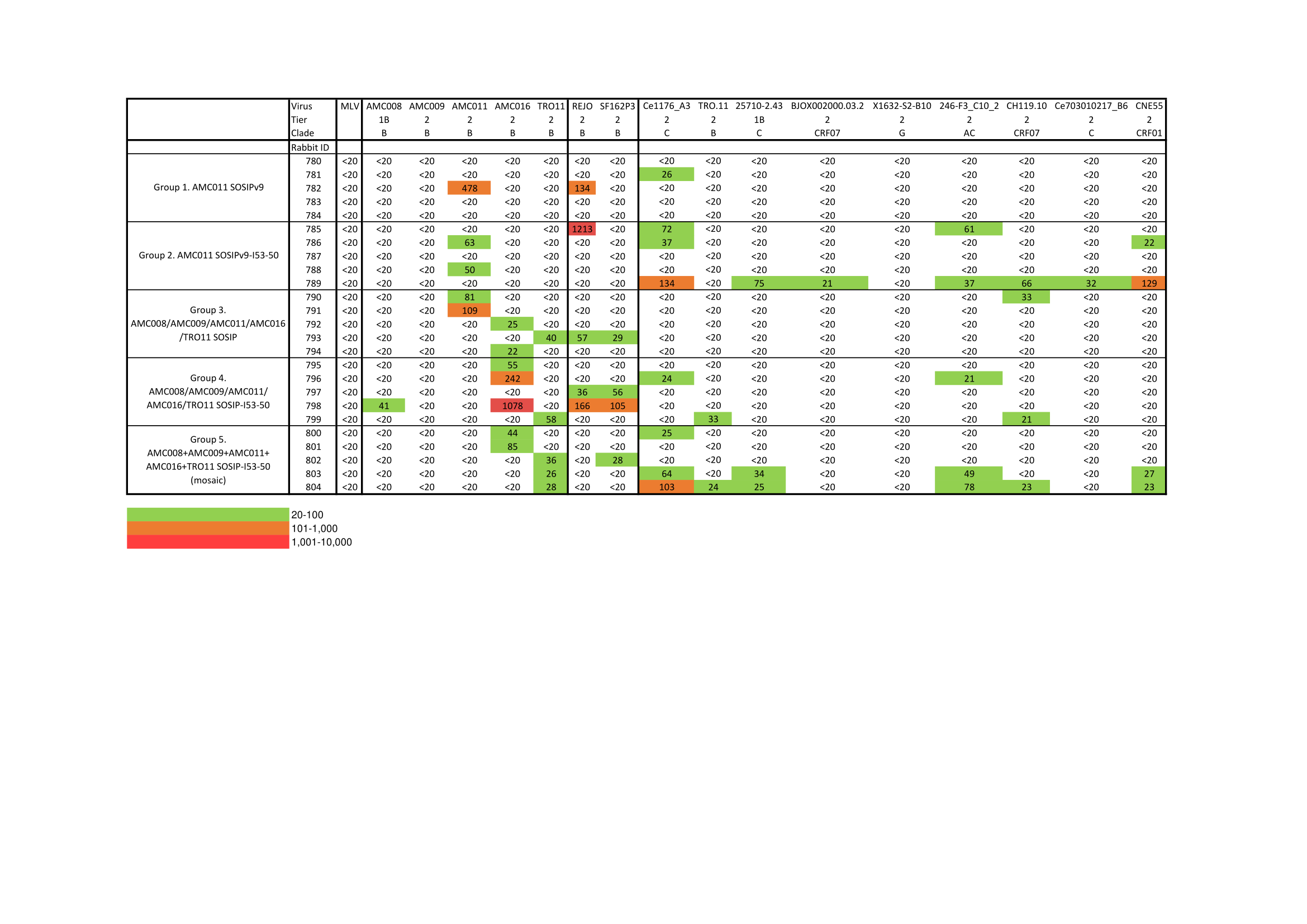

Supplement: S4 Table — (TIFF) [file ppat.1012558.s017.tiff]
